# Using Network-Based Machine Learning to Predict Transcription Factors Involved in Drought Resistance

**DOI:** 10.3389/fgene.2021.652189

**Published:** 2021-06-24

**Authors:** Chirag Gupta, Venkategowda Ramegowda, Supratim Basu, Andy Pereira

**Affiliations:** Department of Crop, Soil, and Environmental Sciences, University of Arkansas, Fayetteville, AR, United States

**Keywords:** rice, oryza, drought, transcription factor, gene regulatory network, machine learning, abiotic stress, R shiny

## Abstract

Gene regulatory networks underpin stress response pathways in plants. However, parsing these networks to prioritize key genes underlying a particular trait is challenging. Here, we have built the Gene Regulation and Association Network (GRAiN) of rice (*Oryza sativa*). GRAiN is an interactive query-based web-platform that allows users to study functional relationships between transcription factors (TFs) and genetic modules underlying abiotic-stress responses. We built GRAiN by applying a combination of different network inference algorithms to publicly available gene expression data. We propose a supervised machine learning framework that complements GRAiN in prioritizing genes that regulate stress signal transduction and modulate gene expression under drought conditions. Our framework converts intricate network connectivity patterns of 2160 TFs into a single drought score. We observed that TFs with the highest drought scores define the functional, structural, and evolutionary characteristics of drought resistance in rice. Our approach accurately predicted the function of OsbHLH148 TF, which we validated using *in vitro* protein-DNA binding assays and mRNA sequencing loss-of-function mutants grown under control and drought stress conditions. Our network and the complementary machine learning strategy lends itself to predicting key regulatory genes underlying other agricultural traits and will assist in the genetic engineering of desirable rice varieties.

## Introduction

The occurrence of environmental stressors, such as extreme drought, heat, cold, and salinity, negatively regulates the growth and development of crop plants, causing a substantial loss in yield and quality (Boyer, 1982; Bray, 1997; Yamaguchi-Shinozaki and Shinozaki, 2006; Palanog et al., 2014). Plants and specific genotypes within a plant species that can withstand sub-optimal growth conditions would be identified as ‘stress-tolerant’ and offer examples to study the mechanisms involved in their survival and productivity in terms of yield. While conventional breeding has been the preferred method of improving stress tolerance in rice and other crops, modern genomics, and genetic engineering strategies have become an integral part of trait enhancement programs (Umezawa et al., 2006; Ashraf, 2010; Gaj et al., 2013). However, a prerequisite for the effective use of genetic engineering tools in trait improvement is the prior knowledge about candidate genes that are likely to produce a desirable phenotype when genetically intervened. Although transcriptome analysis of rice under water-limited conditions, for example, has identified thousands of differentially expressed genes, it is difficult to narrow down the selection of candidate genes for testing function and genetic modification of drought resistance (DR). This lack of candidate genes will be a significant bottleneck in the future, as it impedes our ability to upscale targeted genetic screens in order to select leads for further crop improvement (Gutterson and Zhang, 2004; Century et al., 2008; Jansing et al., 2019; Baxter, 2020). Therefore, new data-driven approaches capable of discovering critical genes regulating complex traits like DR are needed.

Gene regulatory networks (GRN) play a central role in mediating plant responses to environmental changes (Chen and Zhu, 2004; Clauw et al., 2016; Lovell et al., 2018). Transcription Factors (TFs) are vital nodes (genes) in these networks as they regulate the expression of several downstream genes involved in many stress-responsive pathways and biological processes. TFs act as ‘switches’ in genetic networks and can be exploited to engineer stress-resistant crop varieties (Tran et al., 2010; Rabara et al., 2014; Krannich et al., 2015; Wang et al., 2016; Hoang et al., 2017). Such gene activity can be monitored dynamically under varying experimental conditions using genome-scale technologies such as microarrays and RNA-sequencing. Integration of such transcriptome-level datasets for inference of GRNs remains a feasible approach (Razaghi-Moghadam and Nikoloski, 2020). Transcriptome-based network inference techniques have also shown great promise in accelerating *in silico* gene discovery for *in planta* gene validation in plants (Li et al., 2015; Gupta and Pereira, 2019; Haque et al., 2019).

There are several caveats to GRN inference using expression data, which mainly stem from co-expression used as a proxy for co-regulation. A physical interaction (e.g., TF-promoter and TF-TF protein complex) cannot be guaranteed with an observed TF-gene pair that co-express. Incorporating TF-DNA binding data (e.g., ChIP-seq datasets, predictably conserved TF-DNA binding motif relationships) into the network inference workflow can overcome some of these limitations. However, careful methodological considerations can also circumvent some of these limitations. An increasing corpus of network inference algorithms aims to eliminate likely indirect interactions between TFs and other genes, i.e., correlations arising from transcriptional regulation cascades. These algorithms provide an advantage of inferring GRNs using expression data to cover those TFs for which DNA-binding sites have not been found or confirmed as yet, which remains the case for rice (Wilkins et al., 2016), and mostly all crops. We believe that removing TFs with no DNA-binding data from network inference essentially leads to the loss of regulatory signals that can be measured by analyzing expression patterns.

The outcomes of network inference considerably differ between different algorithms because they adopt different statistical assumptions and filtering schemes to detect regulatory interactions in expression patterns. Therefore, different network inference strategies have their strengths and weaknesses, making it difficult to narrow down on a single best approach (Stolovitzky et al., 2009; Marbach et al., 2010). Previously, large-scale evaluations showed that the advantages of combining predictions from different algorithms complement each other, and their limitations tend to cancel out (Michoel et al., 2009; De Smet and Marchal, 2010; Marbach et al., 2012; Hase et al., 2013). Rather than relying on only one approach, an ensemble-centric approach of combining predictions from multiple algorithms appears to be an excellent strategy to infer GRNs even in plants (Vermeirssen et al., 2014; Taylor-Teeples et al., 2015; Redekar et al., 2017; Foo et al., 2018).

Post the inference of a GRN, mining relevant signals that may lead to actionable hypotheses is not straightforward. For example, a typical network analysis workflow aims to find modules (communities of densely connected genes) in the network and assign a biological meaning to these modules using statistical enrichment of gene ontologies and pathway annotations. Biological interpretation using enrichment analysis typically require modules with a considerable number of genes for reliable overlap statistics with the already sparse and incomplete functional annotations. Therefore, modules containing many genes are readily interpreted in functional contexts, while smaller modules typically remain less interpretable.

Large modules of densely connected genes can be un-inviting for experimental biologists who wish to apply network models in the wet-lab. Biologists should have a protocol that converts complex ‘hairballs’ of connected genes into a single score for each gene, allowing non-subjective candidate prioritization before validation. Gene prioritization before experimental testing is vital for reducing associated costs, especially when one intends to work on more than one node in a sub-network (or module) of interest. The popular concept of ‘hub’ genes (genes with a relatively large number of connections in the network) is contextual (Langfelder et al., 2013; Walley et al., 2016; Vandereyken et al., 2018), as hubs in a protein coexpression network can be very different from hubs in a protein coexpression network (Walley et al., 2016). In terms of regulatory networks, studies in yeast have shown that hierarchy, rather than connectivity, better reflects regulators’ importance (Bhardwaj et al., 2010). Therefore, new computational approaches beyond the estimation of ‘hubbiness’ or other network parameters for gene prioritization are required.

Gene prioritization is an essential technique for selecting lead candidates before experimental testing. One might assume that a simple test of differential expression can be used for gene prioritization based on the magnitude of fold change under certain experimental conditions. However, we argue that this method is not the most logical approach for gene prioritization, especially for TFs. Given their regulatory nature, subtle changes in the expression of TFs could have profound effects on the expression of downstream genes. Therefore, technically speaking, such TFs might not naturally qualify to find a position toward the top of the sorted list of genes based on fold changes.

Recently, supervised machine learning has been useful in generating predictive models for various research aspects in plant and crop biology (Ma et al., 2014; Sperschneider, 2019, 2020). Supervised machine learning algorithms leverage experimentally validated gold-standard example genes from the literature to make new predictions on genes with similar attributes. For example, thousands of genomic and evolutionary features that characterize known essential genes were used to train models predictive of other untested lethal-phenotype genes (Lloyd et al., 2015). Similarly, several distinguishing features of genes currently annotated in secondary or primary metabolism pathways were used to train models capable of predicting new specialized metabolism genes (Moore et al., 2019). Putative *cis*-regulatory elements (CREs) involved in general abiotic and biotic stress responses (Zou et al., 2011), and CREs involved in the regulation of root cell type responses to high salinity stress (Uygun et al., 2019) have also been identified by the application of supervised machine learning models.

We are particularly interested in studies that used a genome-scale network, instead of heterogeneous genomic features, as input to the learning algorithm. Such frameworks aim to capture the network connectivity patterns that characterize a set of gold standard (or marker) genes. Network-based machine learning has been used to make reliable predictions on disease-gene associations in humans (Guan et al., 2010, 2012; Krishnan et al., 2016; Liu et al., 2019). Such predictive systems have immense potential in the development of decision systems in clinical diagnostics. However, whether this network-based supervised machine learning approach can be applied to predicting regulatory genes associated with specific agricultural biology traits remains to be tested.

**FIGURE 1:**
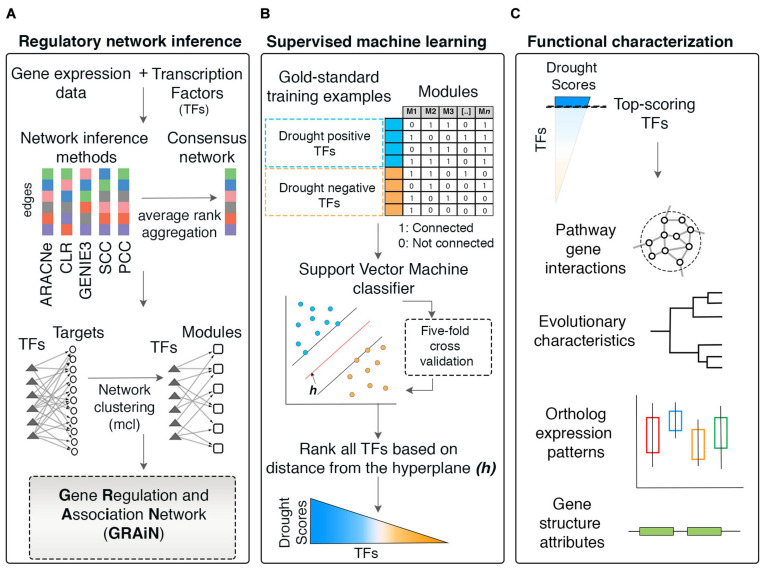
Workflow of the network-based machine learning framework developed in this study. **(A)** A gene regulatory network (GRN) depicting regulatory relationships between transcription factors (TFs) and potential target genes was inferred from large-scale expression data (microarrays) of rice. An ensemble of network prediction algorithms was applied to the data and the networks inferred by different algorithms were statistically combined using the average rank aggregation method, resulting in a single consensus gene regulatory network. This network then was clustered using a network clustering algorithm to identify modules of coregulated, and, therefore, functionally associated genes. This modular core of the network was then reconciled with the GRN core, resulting in weighted assignments of TFs as the potential regulators of the modules. The network was named Gene Regulation and Association Network (GRAiN). mcl, Markov clustering algorithm. **(B)** Several rice knowledgebases and the literature were mined to obtain a list of TFs that are experimentally validated and reported as regulators of drought response phenotypes in rice. We found 165 such TFs reported to date. We regarded these TFs as the ‘gold standard’ examples of drought resistance (DR). All DR TFs were labeled as the ‘drought positive’ class. The group of TFs that did not differentially express in our reanalysis of several published drought experiments were labeled as the drought negative class. These benchmark drought TFs (positive and negative class), along with their network connectivity patterns in GRAiN, were used as input to train a binary classification algorithm, the support vector machine (SVM). The SVM learnt unique network patterns that can discriminate between the two classes of benchmark TFs. These patterns were fivefold cross-validated and subsequently used to predict the class label (positive or negative) of the remaining unlabeled TFs (ones that are neither in the positive nor the negative class). The final model’s output was used to represent each TF in GRAiN (2160 total) along a continuous spectrum (called drought scores), representing its potential association with drought resistance. **(C)** The functional, evolutionary, and genomic features unique to most TFs at the top end of the drought score spectrum were identified and described. GRAiN and predictions on regulators of DR can be freely accessed online at http://rrn.uark.edu/shiny/apps/GRAiN/.

In this study, we developed the Gene Regulation and Association Network (GRAiN) of rice. We built GRAiN using a collection of publicly available gene expression datasets and an ensemble of five different network prediction algorithms (Figure 1A). GRAiN links 2160 rice TFs to 740 modules of co-regulated genes that manifest under abiotic-stress conditions. We utilized GRAiN to develop a model predictive of TFs involved in the regulation of DR. We used a training set of TFs that are already known regulators of DR as input to a learning algorithm (support vector machine). The learning algorithm used this training data to learn general network patterns that characterize DR. We then used the trained model to identify other TFs that resemble TFs in the training set. Our strategy scored 2160 rice TFs according to their potential association with DR (Figure 1B). Leveraging these scores, we described the functional, evolutionary, and structural characteristics of drought regulation (Figure 1C). We also developed a web application to browse GRAiN^1^ easily. Furthermore, we experimentally validated GRAiN’s predictions on the OsbHLH148 TF using *in vitro* protein-DNA binding assays and mRNA sequencing loss-of-function mutants grown under control and drought stress conditions. Our study will provide a valuable resource for generating new testable hypotheses on the genetic basis of stress tolerance in rice.

## Results and Discussion

We obtained 35 independently published publicly available gene expression datasets. These datasets comprise samples from 50 different genotypes and cultivars, three developmental stages of rice growth, five different tissues, and nine different environmental stress conditions. We normalized and integrated the datasets to create a single gene expression matrix representing 35,151 rice genes’ intrinsic expression in 265 individual samples. Our objective was to utilize the correlated and mutually informative expression patterns in this matrix to predict potential regulatory interactions between TFs and target genes.

### The Outcome of Network Inference Varies Between Different Algorithms

Rather than using a single algorithm for the inference of the rice GRN, we created an ensemble of five diverse methods that use different edge-scoring and filtering strategies. We included Context Likelihood of Relatedness (CLR) and Algorithm for Reconstruction of Accurate Cellular Networks (ARACNe) in the first category of algorithms that use mutual information (MI) to estimate similarity in expression patterns. We included Pearson’s Correlation Coefficient (PCC) and Spearman’s Correlation Coefficient (SCC) as the second category’s two correlation-based methods. In the third category, we used GEne Network Inference by an Ensemble of trees (GENIE3) algorithm as the regression-based method that infers edges with directionality. We then supplied each of these five algorithms with the gene expression matrix to predict regulatory interactions (edges) between TFs and target genes (see section “Materials and Methods”).

**FIGURE 2:**
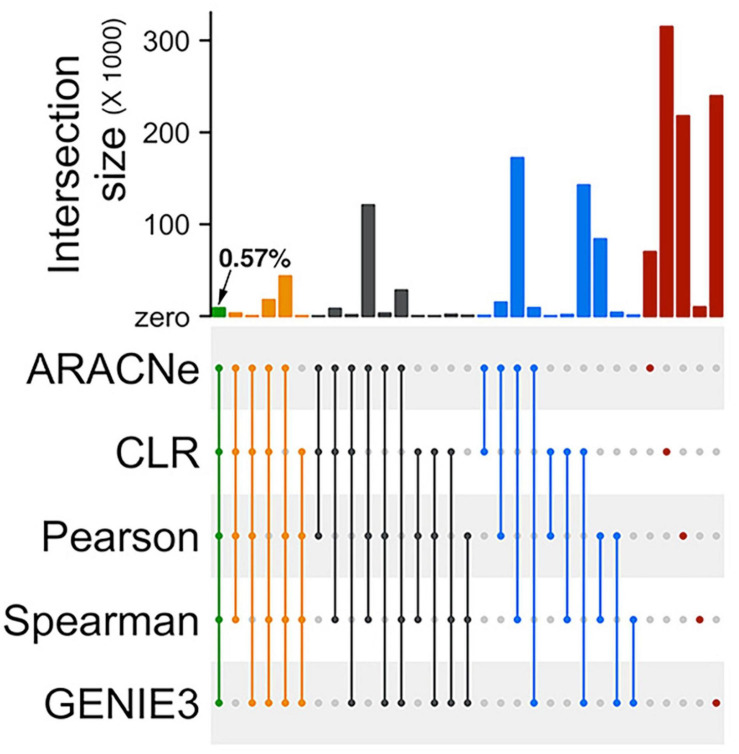
Low overlaps between edges inferred by different network prediction algorithms. An Upset plot showing overlaps between the two mutual-information based algorithms (ARACNe and CLR), two correlation-based algorithms (PCC and SCC), and one decision-tree based algorithm (GENIE3) used for prediction the rice gene regulatory network. ARACNe, Algorithm for Reconstruction of Accurate Cellular Networks; CLR, Context Likelihood of Relatedness; PCC, Pearson’s Correlation Coefficient; SCC, Spearman’s Correlation Coefficient; and GENIE3, Gene Network Inference by an Ensemble of trees. The filled dots in the canter matrix indicate association between the respective sets and the bars on the top show size of the intersection. Green, orange, black, and blue bars indicate intersection size between five, four, three, and two algorithms. Red bars indicate unique edges identified by the corresponding algorithm.

We retained only the top 500,000 high confidence edges from each algorithm’s outcome to reduce the computational burden in the network analysis workflow (Supplementary Data 1). These 500,000 edges represented less than 1% of all theoretically possible edges between TFs and their target genes in the input gene expression matrix (see section “Materials and Methods”). We asked if these high confidence edges predicted by the five algorithms are similar. We observed a minimal overlap (less than 1%) between all five algorithms’ outcomes (Figure 2). The most considerable fraction of unique edges came from the CLR algorithm, followed by GENIE3 and PCC. We observed a relatively more extensive overlap between the algorithms in different categories than algorithms in the same category. For example, the overlap between SCC and ARACNe eclipses the overlap between SCC and PCC. This is probably because SCC and ARACNe, unlike PCC, are not constrained to detecting only linear correlations between TF and target genes. Similarly, we observed a relatively more generous overlap between GENIE3 and CLR than between CLR and ARACNe. This could be because, for filtering edges, both CLR and GENIE3 account for each gene’s local distribution of background values separately. On the other hand, ARACNe examines triplets of connected genes and relies on a global threshold to eliminate the edge with the lowest score in each triplet as an indirect relationship.

Overall, our analysis suggests that the outputs of different network inference algorithms vary greatly and depend mainly on the filtering schemes used to eliminate low confidence edges.

### The Performance of Network Inference Can Be Improved by Combining Networks Inferred by Multiple Algorithms

**FIGURE 3:**
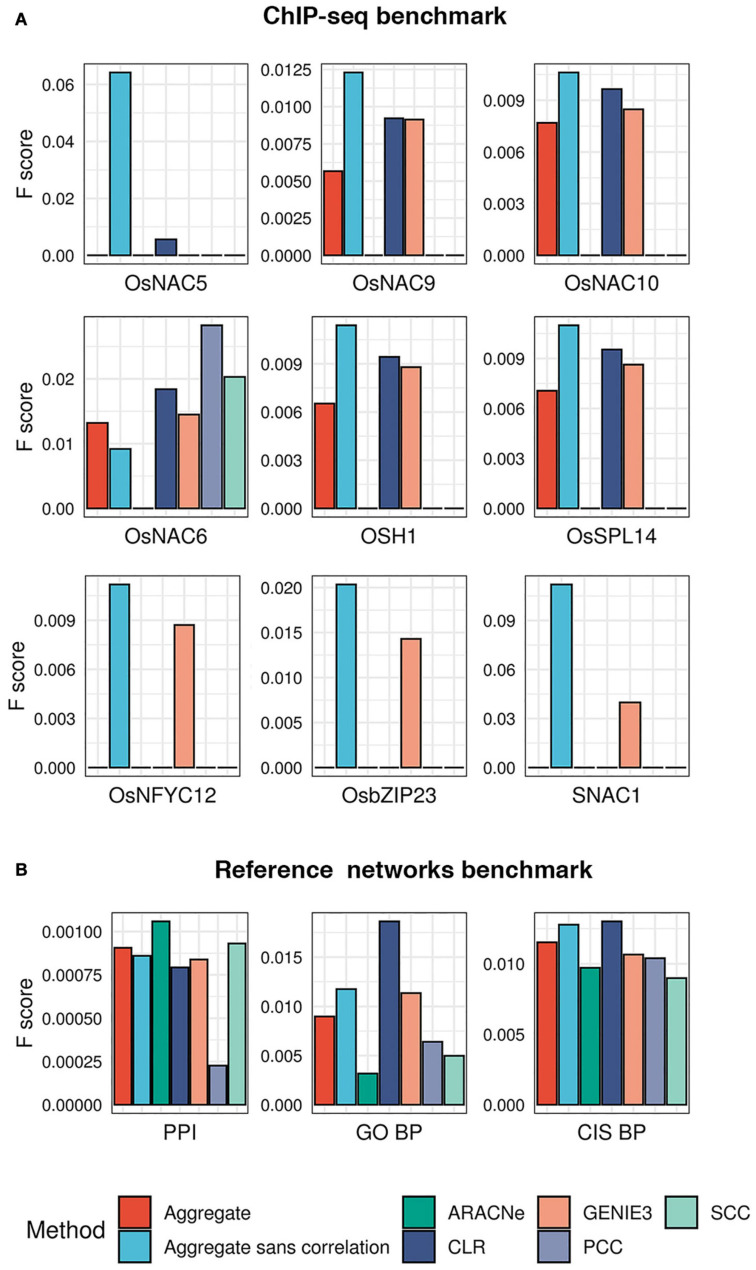
Evaluation of the five network prediction algorithms and their aggregate. **(A)** A ChIP-seq benchmark for 9 TFs was created from publicly available datasets. For each of these 9 TFs, we checked the overlap between experimentally validated targets (ChIP-bound genes) and network-predicted targets (genes predicted by each of the five algorithms in our ensemble). This evaluation was also made for the consensus network obtained by statistically aggregating the predictions from the five algorithms. Each bar plot shows *F1* scores (*y* axis; a measure of performance, the higher the better) of each algorithm (*x* axis) in correctly predicting ChIP-targets of TFs. **(B)** Due to the unavailability of experimentally validated targets of a large number of TFs in our network, we created additional ‘reference networks’ to gauge the quality of the inferred networks. PPI, reference network derived from the predicted protein–protein interaction network of rice (PRIN database); GO BP, reference network derived from co-annotations in select gene ontology biological process terms; and CIS BP, reference network obtained by utilizing the available putative DNA-binding sites of TFs in the CIS BP database (see “Materials and Methods” for details). The bar plots of *F1* scores shows the performance of each algorithm in reconstructing the reference networks.

To test the performance of each algorithm in predicting known targets of TFs, we obtained experimentally identified targets of 9 TFs in published ChIP-seq experiments (Lu et al., 2013; Tsuda et al., 2014; Zong et al., 2016; Chung et al., 2018; Li et al., 2019). Using these 9 TFs as the benchmark, we asked what fraction of their ChIP targets each algorithm could correctly predict. We observed that GENIE3 recovered ChIP targets of 8 out of the 9 TFs in the benchmark, CLR recovered targets of 6 TFs, while ARACNe, PCC, and SCC recovered targets of only 1 TF each (Figure 3A and Supplementary Table 1). To quantify each algorithm’s overall performance as a single measure, we calculated the *F1* score as the harmonic mean of precision (the fraction of predicted targets that are also ChIP targets) and recall (the fraction of ChIP targets amongst all predicted targets). We observed that the CLR algorithm consistently achieved the highest *F1* score in more cases than the next best performer, GENIE3 (Figure 3A).

Note that the TFs used in the ChIP-seq benchmark represents only a fraction (less than 1%) of all TFs for which targets were predicted. Therefore, we could not regard the ChIP-seq dataset as a comprehensive benchmark for evaluating different network inference methods we used in our study. We built additional *ad hoc* ‘reference networks’ to gauge the algorithms’ performance. We sought to create reference networks that reflect putative targets of TFs that can be predicted independently of their expression profiles, since we built the network using only expression data.

For the first reference network, we obtained experimentally verified protein interaction partners from the protein interaction network of rice (PRIN) database (Gu et al., 2011). For the second reference network, we used promoters of genes with known DNA-binding sites of TFs by analyzing the CIS-BP database (Weirauch et al., 2014). We created the third reference network by linking TFs and non-TFs if they are co-annotated in carefully selected, non-redundant Gene Ontology (GO) Biological Process (BP) terms. For the GO BP reference network, we assumed the TF and non-TF genes co-annotated to the same BP terms are more likely to have a biological relationship, relative to genes annotated to distant or unrelated GO BP categories. Although the second and the third reference networks do not guarantee real biological relationships between TFs and target genes, they provided us with a valuable resource to include more TFs in the evaluation and gauge the agreement between different data types in predicting targets of TFs.

We asked what fraction of edges present in the three independent reference networks could be predicted by each algorithm in our ensemble. We observed that the CLR algorithm consistently attained the best recall rate in all three reference networks and outperformed other methods in reconstructing the CIS-BP and GO BP reference networks in terms of the *F1* score (Supplementary Table 1). The ARACNe algorithm performed with the best precision in the CIS-BP reference network and outperformed others in reconstructing the protein interaction network in terms of the *F1* score. SCC’s precision in predicting CIS-BP edges was lower than that of ARACNe but better than the other three methods. We observed that PCC outperformed SCC in CIS-BP and GO BP reference networks (Figure 3B).

Based on these evaluations, we could not establish any single algorithm as the best performer in reconstructing the three reference networks or correctly predicting ChIP targets. Therefore, we asked whether combining the networks inferred by different algorithms into a single network improves the overall performance. Rather than taking a union or an intersection, we chose the ‘average rank aggregation’ approach to combine different networks (Marbach et al., 2012). The underlying idea behind the average rank aggregation approach is that a biologically meaningful edge tends to occur consistently at high ranks (or confidence scores) across different networks predicted using different approaches. Hence, averaging the ranks of individual edges essentially reinforces likely real edges in the final aggregate network. This approach has been previously shown to efficiently integrate different edge-weighted GRNs into a single consensus network, even in plants (Vermeirssen et al., 2014).

Following the average rank aggregation method, we combined the networks inferred by all five algorithms in our ensemble into a single GRN (see section “Materials and Methods*”*). Then, we asked whether this aggregation improved the accuracy by re-evaluating the ChIP-seq benchmark and the three reference networks described above. We observed that the aggregate network could not outperform CLR and GENIE3 in most cases in the Chip-seq set but consistently outperformed ARACNe, SCC, and PCC (Figure 3A). Interestingly, removing the two correlation-based methods from the aggregate almost always improved the performance, compared to the aggregate that included the correlation-based methods (Figures 3A,B). The aggregate-sans-correlation network achieved, on average, 49 and 20% increase in *F1* score when tested on the ChIP-seq benchmark and the three reference networks, respectively, relative to the aggregate that included PCC and SCC. Therefore, the aggregate of CLR, ARACNe, and GENIE3 was chosen as the final ‘consensus’ GRN of rice and used in further analysis.

### Clustering the GRN Identifies Modules of Functionally Related and Co-regulated Genes

Our next objective was to find clusters of co-regulated genes, i.e., groups of genes regulated by the same set of TFs. Assuming a guilt-by-association, we expected network clustering to identify modules of co-regulated, and therefore functionally related genes. Such modules thereby provide pointers on pathways and biological processes that could be under the regulatory control of specific TFs (Hartwell et al., 1999; Segal et al., 2003; Ma et al., 2004; Joshi et al., 2009). To achieve such a network clustering, we first linked target genes that had high overlaps between their predicted regulators in the consensus GRN, as done previously with the Arabidopsis stress GRN (Vermeirssen et al., 2014). We then applied the Markov clustering algorithm to this co-regulated gene network (van Dongen and Abreu-Goodger, 2012). We identified a total of 740 modules, with an average of 45 genes each (Supplementary Data 2).

To confirm the regulatory association of genes within each module, we analyzed their 1000 bp upstream promoter regions to check whether putative DNA-binding sites were over-represented. We employed the FIRE (finding informative regulatory elements) algorithm (Elemento et al., 2007). FIRE uses a *de novo* approach to find short stretches of DNA-sequence motifs that explain promoters’ module-membership (Elemento et al., 2007). Application of the FIRE algorithm on our network data detected 84 DNA-binding motifs within the co-regulated modules. We observed that more than 50% of all coregulated modules harbor between five and ten motifs each (Supplementary Figure 1A), indicating a high level of coordination between TFs. We observed that eighty-one of the FIRE-detected motifs are identical to known plant CREs listed in multiple plant databases and other sources, whereas three are novel DNA motifs (Supplementary Figure 1B and Supplementary Data 3). Network analysis of the genes with the three novel motifs suggests that two distinct groups of TF families target them (Supplementary Figure 2). Overall, the over-representation of common plant CREs in the promoters of module genes testified that the observed modules are non-random gene groupings and represent sets of co-regulated genes.

To further test whether the observed gene modules also represent a joint biological function, we used function annotations from the rice GO BP category and pathway-level annotations from Mapman, KEGG, and CYC databases. We found statistically significant associations of these functional annotations in 31% of all observed modules (hypergeometric test FDR corrected *p-values* < 0.05). We also found that ∼41% of all modules we detected in this study were preserved in an independent coexpression network we built earlier with a different dataset and the cluster detection algorithm (Krishnan et al., 2017). Interestingly, 22% of these preserved modules are the ones that could not be annotated by gleaning function annotation databases, highlighting significant gaps still exist in the current state of function annotations of rice genes (Supplementary Data 4).

We linked TFs to the co-regulated modules, and set the edge-weight according to the Jaccard’s Index (JI) of overlap between the predicted targets of TFs in the consensus GRN and module genes. The JI ranges between 0 and 1, where 0 indicates no regulatory association between the corresponding TF-module pair and a JI of 1 indicates a certainly likely regulatory association. Therefore, these operations generated a modular GRN of rice, where TFs are directly linked to target genes and indirectly but quantifiably associated with functional processes. We refer to this network as GRAiN. GRAiN can be searched through an online portal (demonstrated in the last section of this manuscript).

### Sorting Genes Based on the Magnitude of Differential Expression Is Not a Suitable Approach for Gene Prioritization

Past genetic research in rice has revealed several examples of ‘gold-standard’ drought genes identified by reverse-genetics. This documented knowledge about the genetic basis of drought is most comprehensive among other abiotic-stresses. It presents us with a unique opportunity to test whether differential expression measures can be used as a proxy for gene prioritization. We scanned the functional rice gene database (Yao et al., 2018), the rice mutant database (Zhang et al., 2006), and the Oryzabase (Kurata and Yamazaki, 2006). We found 732 genes with genetic evidence of association with drought listed in these knowledgebases. This list of ‘drought associated’ genes did not represent any particular physiological, morphological, or biochemical phenotype typically measured in the analysis of drought response. For the sake of convention, we use ‘DR’ as a broad term to encapsulate various molecular mechanisms by which plants adapt, escape, or otherwise tolerate water limiting conditions (Levitt, 1980; Basu et al., 2016). There are currently 165 DR TFs in this list of known drought genes. We regarded these 165 TFs as the gold-standard examples of DR, and refer to them as DR TFs.

**FIGURE 4:**
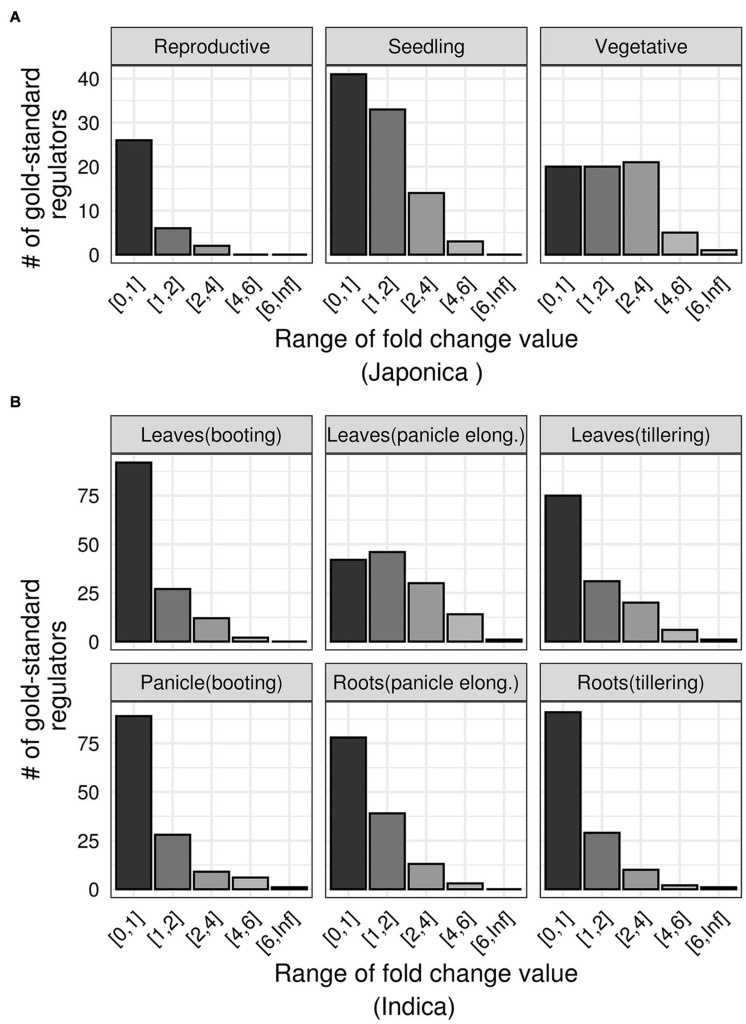
Differential expression patterns of gold standard drought regulators. **(A)** The range of absolute fold-change values (*x-axis*) of gold standard TFs (*y-axis*) in three growth stages of *Japonica* rice variety exposed to drought (data from GSE81253). **(B)** The range of absolute fold-change values (*x-axis*) of gold standard TFs (*y-axis*) in multiple tissues of *indica* rice variety exposed to drought (data from GSE26280).

Next, we asked how the DR TFs respond to drought in terms of differential expression. We re-analyzed data from seven independently published drought experiments performed on multiple varieties, growth stages, and tissues of rice plants (Wang et al., 2011; Ding et al., 2013; Pabuayon et al., 2016; Mishra et al., 2018). We estimated genome-wide fold change values in each of these datasets, and ranked all TFs based on the absolute values of these fold changes. We observed that, in each experiment, the majority of the DR TFs showed minimal changes (fold change values < 1) regardless of the tissue, growth stage, or the variety of rice plant (Figures 4A,B). This suggests that gene prioritization based on differential expression values is constrained by experimental factors and will downplay those that show subtle changes in expression but have relevant biological effects. Therefore, we need a more sophisticated technique for prioritizing TFs that likely regulate drought responses in rice.

### Network-Based Supervised Machine Learning Enables Classification and Scoring of Drought Resistance Regulators

Our next objective was to develop a network-based gene prioritization framework that can be objectively tested using independent data. We utilized the two good pieces of information at hand; a high-quality modular GRN (GRAiN) and a list of literature curated gold-standard DR TFs. We posited that advanced machine learning models could be trained to recognize network patterns in GRAiN that characterize the gold standard DR TFs. These patterns could then be matched with the patterns of other yet untested TFs and estimate whether they resemble the DR TFs.

**FIGURE 5:**
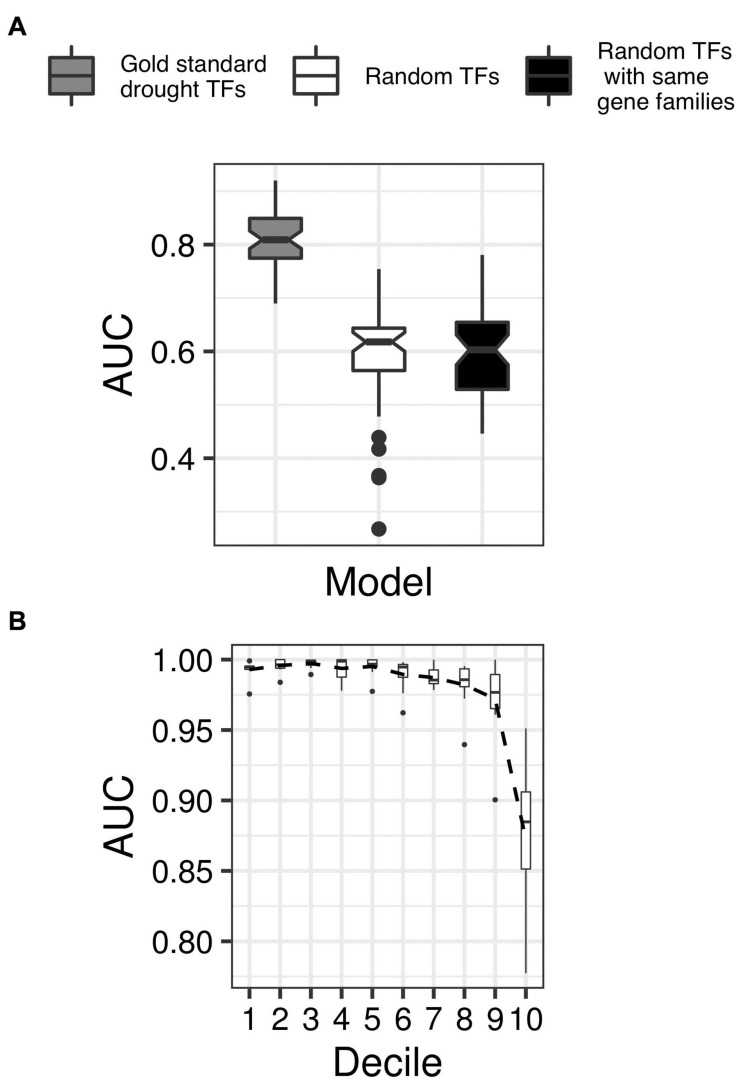
Cross-validation of the network-based classifier. **(A)** Boxplots showing the distribution of the area under the precision-recall curve (AUC-PR; *y-axis*) in ten independent runs of fivefold cross-validation tests of the classifier trained using gold standard drought TFs (shaded gray), the classifier trained using randomly picked TFs instead of gold standard TFs (shaded white), and the classifier trained using randomly chosen TFs but from the same families like that of the gold standard examples (shaded black). The non-overlapping notches in the boxplots indicate significant differences in the median AUC-PR for all three classifiers. **(B)** TFs were sorted according to their decreasing order of drought scores assigned by the final classifier and grouped into 100 equal-sized bins. Expression levels (transcript per million units) of TFs in each bin were then used as features to classify a set of labeled RNA-seq samples as drought or control (data from GSE74793). Each boxplot shows the distribution of AUC-ROC *(x-axis)* from threefold cross-validation tests in groups of ten bins, with lower-numbered bins (*y-axis*) indicating TFs with higher drought scores. The black dotted line connects the mean of each decile’s AUC-ROC scores, indicating decreasing AUC-ROC with lower drought scores.

We chose the support vector machine (SVM), a popular binary classification algorithm (Cortes and Vapnik, 1995), to develop the DR classifier. We supplied the SVM with a training set of TFs and their connectivity patterns in GRAiN, along with binary labels indicating whether each TF is a DR TF or not (see section “Materials and Methods”). We evaluated the SVM’s accuracy using fivefold cross-validation tests and the area under the precision-recall curve (AUC-PR) statistics. The AUC-PR ranges between 0 and 1, with values closer to 1 indicating the model’s superior performance. Our DR classifier achieved an average AUC-PR of 0.81 in 10 independent runs of five-fold cross-validation tests. We asked if this AUC-PR could be achieved by randomly picking TFs from the rice genome instead of using the DR TFs for training. We found the DR classifier’s AUC-PR to be significantly larger than the AUC-PR of the classifier trained using randomly picked TFs. Because family membership could play an essential role in TF function, we also tested the AUC-PR of the classifier trained by randomly picking TFs while maintaining the family distribution as that of the DR TFs. We observed that the AUC-PR of this classifier was not different than the random classifier, indicating that family memberships of TFs is not indicative of their roles under drought (Figure 5A).

We applied the cross-validated SVM model to the whole network of 2160 TFs. We used the model’s output – which represented the model’s confidence in its classification of a TF as a DR TF – to rank each TF. We then scaled the ranks within a range of 0 and 1 to make the ranks more interpretable, and referred to the resulting scores as drought scores (DS). The TFs with DS close to 1 have the strongest predicted association with DR, while TFs with relatively smaller DS values are less likely to be associated with DR (Supplementary Data 5).

To evaluate this scoring scheme objectively, we asked if the occurrence of drought can be inferred by the transcript abundance of TFs with the largest DS. In other words, we wanted to check if the intrinsic expression levels of TFs with high DS can indicate if a plant has sensed drought or not. Operationally, this technique is similar to the ones used in developing clinical diagnostic models that seek to classify human patient samples as disease or healthy based on the expression levels of marker genes. To perform such an evaluation of our model, we downloaded and reanalyzed the recently published RNA-seq dataset of 214 seedling samples (71 drought samples and 143 control samples) from four different rice varieties (Wilkins et al., 2016). Assuming the first decile TFs in our predictions as ‘drought markers’, we asked whether the intrinsic expression levels (measured as transcripts per million units) of these drought markers can predict a sample in the Wilkins dataset as control or drought.

We observed that RNA-seq sample classification accuracy was almost perfect when we used the intrinsic expression of top decile TFs as features. However, this accuracy gradually decreased as we moved toward lower decile TFs (Figure 5B). We observed that the expression levels of TFs in the last decile was least accurate in classifying a sample as drought or control (Figure 5B). This analysis suggests that the top-scoring TFs are likely responsible for causing the transcriptional-level changes that occur under drought, and therefore validates our ranking approach.

### Predicted Regulators of Drought Resistance Are Involved in Hormone-Mediated Responses

It is important to note that the DS we predicted and the out-degree of TFs in the network are not correlated (Supplementary Figure 3), indicating that the predicted DS do not merely reflect on the ‘hubbiness’ of TFs. We investigated the few modules (features) that served as the best predictors for the classification of DR TFs in our model. We selected top ‘drought modules’ using the ‘feature importance’ scores from the model output (Supplementary Data 6). We extracted all TF and CREs linked with these drought modules and explored the interconnected network in Cytoscape (Shannon et al., 2003; Supplementary Figure 4).

**FIGURE 6:**
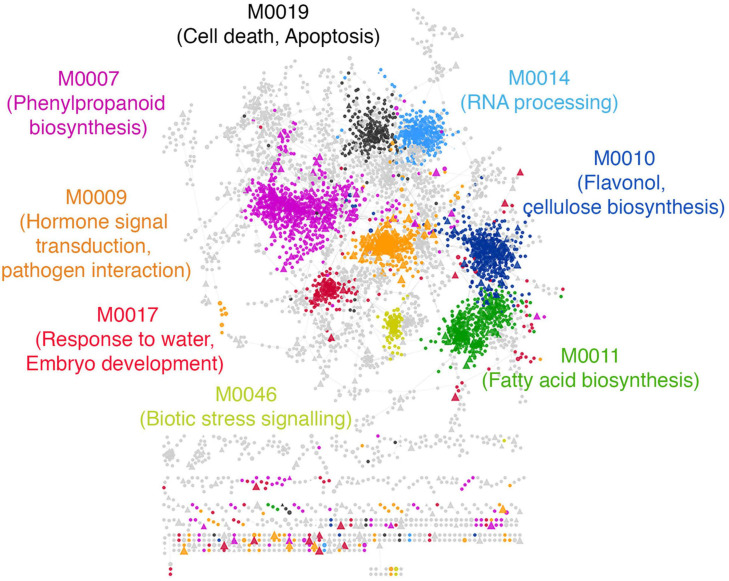
Functional characterization of predicted drought resistance transcription factors. A subset of modules with the highest feature importance scores from the drought classifier were extracted and labeled as ‘drought modules.’ The drought modules consist of a total of ∼6000 genes. The network shows the top 5% edges induced between them. Every circle is a functional gene, and triangles are TFs. Genes within a module are similarly colored, and the GO BP enriched within each module is labeled with the same color in the text. Modules with no statistically enriched GO BP terms are colored gray.

Exploring this network, we found that the drought modules comprise a total of 6968 genes that form core communities enriched in several stress response pathways and biological processes (Figure 6). Interestingly, we found that the drought module are enriched with genes annotated to secondary metabolism pathways broadly related to hormonal signal transduction, such as phenylpropanoid biosynthesis and jasmonic acid biosynthesis. These are traits specific to land plants and is believed to have played an essential role in the adaption of plants to water limiting environments (Kenrick and Crane, 1997; Emiliani et al., 2009; Wang et al., 2015; Ahammed et al., 2016; Verma et al., 2016), given its role in lignin biosynthesis (Fraser and Chapple, 2011). Other relevant GO biological process terms such as ‘response to water,’ ‘response to abscisic acid stimulus,’ ‘cellulose biosynthesis,’ ‘flavonol biosynthesis,’ and ‘trehalose biosynthesis’ were also recovered within the drought modules.

We found that the most prominent *de novo* predicted CREs within the drought modules are related to the abscisic acid response complex ABRE3HVA22 (Shen et al., 1996) and the vascular-specific motif ACIIPVPAL2 (Hatton et al., 1995), along with the light-responsive GT-1 motif (Lam and Chua, 1990), the anaerobic-responsive motif GCBP2ZMGAPC4 (Geffers et al., 2000) and the dehydration responsive DREB1A motif (Maruyama et al., 2004; Supplementary Data 3).

### Predicted Drought Scores Are Associated With Evolutionary Features

The enrichment of genes related to the abscisic acid and salicylic acid pathways, along with jasmonate signaling pathways, as well as some of the observed CREs (e.g., vascular-specific ACIIPVPAL2) within the drought modules indicated a drought response machinery in rice ubiquitous and specific to land plants (Wang et al., 2015). Therefore, we pursued this lead and examined if the orthologs of rice TFs with high DS in our study have conserved responses to drought exposure.

**FIGURE 7:**
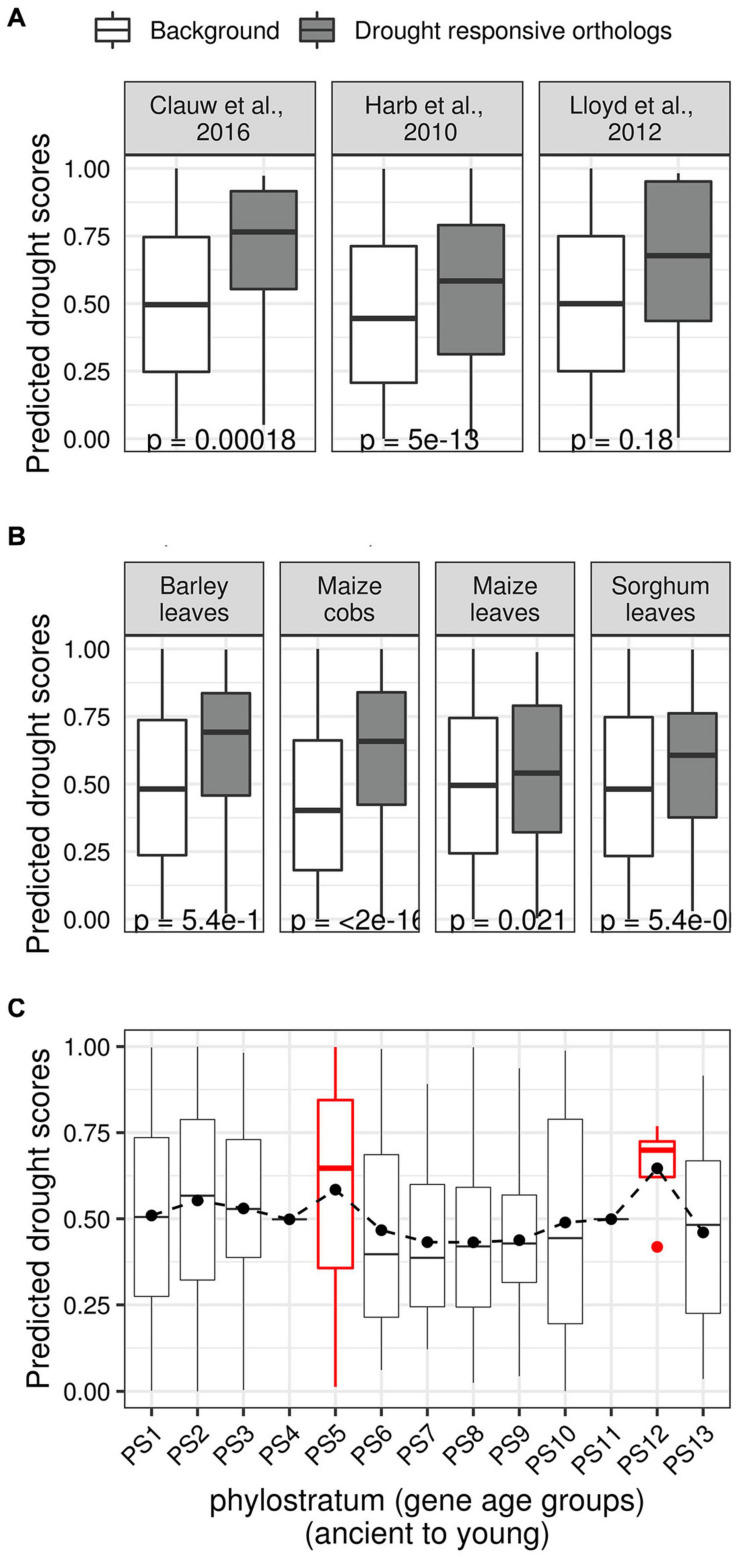
Relationships between predicted drought scores and evolutionary features. **(A)** Three sets of putative drought regulators in Arabidopsis were curated from the literature, and their rice orthologs were identified. The three sets represent rice TFs with orthologs in Arabidopsis genes that were predicted as drought regulators (Clauw et al., 2016), that differentially expressed upon different drought treatment regimes (Harb et al., 2010), have been experimentally characterized as drought regulators (Lloyd and Meinke, 2012). The boxplots show the distribution of the predicted drought scores of these ortholog sets (gray) along with the drought scores of the background of remaining TFs (white; rice TFs that did not become part of the three ortholog sets). In each case, the median predicted drought scores of orthologous rice TFs was found to be significantly higher than the drought scores of the background. **(B)** Similarly, boxplots showing the distribution of drought scores of rice TFs with orthologs in genes that are differentially expressed in different crop datasets. **(C)** Box plot showing the distribution of drought scores in different age groups (ancient to young) according to NCBI taxonomic classification. The distribution of drought scores stays relatively flat, except for two peaks that correspond to the Embryophytes clade (PS5) and the Oryza clade (PS12).

We created three sets of Arabidopsis drought TFs with known orthologs in rice. The first set was differentially expressed TFs in response to mild and severe drought stress we reported previously (Harb et al., 2010). The second set comprised experimentally verified drought TFs in the Arabidopsis phenotype database (Lloyd and Meinke, 2012). The third list of TFs was previously predicted to be involved in mild drought responses (Clauw et al., 2016). We asked if the rice orthologs of these three sets of Arabidopsis TFs have higher DS than the background of all remaining TFs that did not become a part of the three sets (either due to biological variability or due to lack of ortholog identity). In all three sets, we observed a significantly larger mean DS of orthologous TFs compared to the background (Figure 7A). Similarly, we observed that rice TFs with orthologs that differentially expressed in response to the application of drought stress in cobs and leaves of maize (Kakumanu et al., 2012), leaves of barley (Cantalapiedra et al., 2017), and leaves of sorghum, have significantly larger mean DS than the mean DS of the background (Figure 7B).

We also asked if the predicted DS and evolutionary age of a TF are related. We first ordered all rice genes in 13 age groups (phylostrata) starting from the oldest (i.e., genes conserved across all cellular life) to the youngest (i.e., genes that appeared in the terminal clade *Oryza*) (Wang et al., 2018). Plotting the distribution of DS of TFs within each phylostrata (PS) showed two prominent peaks. The first peak in PS5, which corresponds with the Embryophytes (land plants) clade, and the second peak in PS12, which coincides with the Oryza clade, both mirror significant events in the evolutionary history of rice (Figure 7C). We also examined the available pan-genome of rice (Sun et al., 2017) to investigate the distribution of DS of TFs that arose in the terminal clade (*O. sativa*, closely related rice varieties). However, we did not find any significant differences in DS between core and distributed TFs, or TFs that are *Indica*- or *Japonica*-dominant (Supplementary Figure 5).

Overall, our analysis suggests that a large fraction of high-scoring DR TFs possibly played a crucial role in driving critical adaptations of land plants. A few high-scoring TFs that emerged specifically in rice might be involved in recent morphological adaptions that contribute to DR (e.g., panicle architecture, pollen and seed development). Therefore, it would be interesting to analyze the drought phenotypes of mutants lacking these high-scoring TFs specific to rice.

### Predicted Drought Scores and Structural Characteristics Are Related

Recent studies in rice and other organisms suggest that younger genes have relatively simple exon/intron and protein structure (Neme and Tautz, 2013; Cui et al., 2015; Wang et al., 2018). Other studies have shown that simple genes, for example, those that lack introns, are rapidly regulated (Jeffares et al., 2008; Speth et al., 2018). Such genes represent an essential component of the possibly conserved stress response machinery in land plants (Jeffares et al., 2008; Zhu et al., 2016; Morozov and Solovyev, 2019).

**FIGURE 8:**
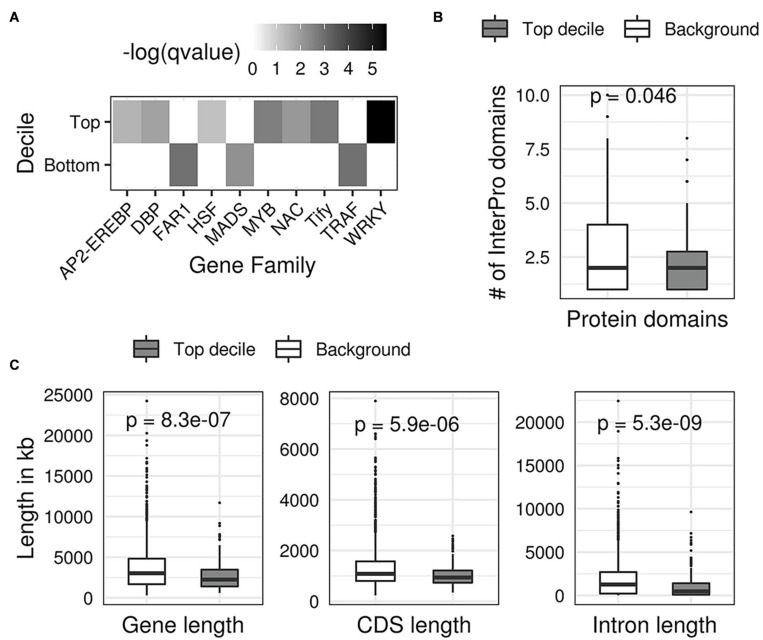
Structural features of transcription factors with the highest drought scores. **(A)** A heatmap showing the enrichment of gene families in the first and last decile TFs (top 10% and bottom 10% drought scores). Each grid in the heatmap shows the FDR corrected -log (*p-*value, Fisher’s exact test) of the gene family on the *x-axis* for the decile on the *y* axis. **(B)** Boxplots showing that top decile TFs contain a significantly different number of protein domains compared to the background of TFs in the remaining deciles. **(C)** Boxplots showing that the top decile TFs have significantly smaller average gene length, coding sequence length (CDS), and intron length compared with the background of remaining TFs.

Following this lead, we next investigated if the predicted DS of TFs and their structural attributes are related since a large fraction of high-scoring TFs our analysis also appear to have first emerged in land plants. We started with examining the family memberships of TFs. We found a statistically significant enrichment of WRKY, Tify, NAC, MYB, and AP2/ERF families among the top 10% TFs with the largest DS (Top decile; FDR corrected hypergeometric test *p* values < 0.1) (Figure 8A). These gene families are well-known to associate with drought stress in multiple crops (Yu et al., 2012; Gahlaut et al., 2016; Hoang et al., 2017). In contrast, we found that TFs with the smallest DS (bottom decile) are enriched in growth and development associated gene families such as the MADS, FAR1, and TRAF (Smaczniak et al., 2012; Tedeschi et al., 2017; Ma and Li, 2018). We observed the top decile TFs (top 10% TFs with highest DS) have relatively fewer InterPro protein domain annotations than the background of all TFs in the remaining deciles (remaining 90% TFs with relatively smaller DS) (Figure 8B). We also observed that the top decile TFs have significantly smaller average gene length, average CDS length, and average intron length (Figure 8C) compared to the background of remaining deciles. This indicates that TFs with high DS are small genes with simple structures.

Overall, our analysis suggests that TFs that likely regulate DR in rice have peculiar functional, structural, and evolutionary characteristics. It is interesting to see such grouping in our data, given that the underlying network using which we made our predictions started with unclassified gene expression data.

### The GRAiN Web Application Is for Experimental Rice Biologists; Using OsbHLH148 as an Example

We used the R Shiny framework to develop a user-friendly web application that allows users to interact with GRAiN and predictions on DR TFs. There are currently two main features of the GRAiN web application active at http://rrn.uark.edu/shiny/apps/GRAiN/. It allows users to search for a single TF gene of interest. In this case, the GRAiN algorithm first retrieves all the genes predicted as targets of the query TF and then uses the inbuilt enrichment analysis tool to find pathways and biological processes over-represented in the predicted targets. The second feature of the GRAiN application allows users to query a set of genes instead of a single TF. In this case, the enrichment analysis tool is used to find co-regulated modules (defined in this study) over-represented in the query genes. Significantly enriched modules are presented back to the user, along with functional (GO BP and Mapman annotations) and cis-regulatory annotations (FIRE-identified CREs and weighted links to TFs).

We chose the rice transcription factor OsbHLH148 (LOC_Os03g53020) to demonstrate the GRAiN web application features. OsbHLH148 was initially present in our list of gold standard drought regulators, as it was earlier reported to be involved in the regulation of drought response via the jasmonic acid pathway (Seo et al., 2011). However, instead of using it as a DR TF in the training set, we kept it a hidden example and treated it as an unlabeled TF throughout model training and evaluation. Since OsbHLH148 was already being studied in our laboratory, our intention behind removing it from the training data was to repurpose its phenotypic and RNA-seq data for experimental validations of the GRAiN web application and the DR classifier.

Our model strongly predicted the association of OsbHLH148 to DR, assigning it a DS of 0.99 and placing it at rank # 4 among all rice TFs. We asked if the GRAiN web application can recover the known functional associations of OsbHLH148. The GRAiN query shows 385 genes predicted as targets of OsbHLH148 (Supplementary Data 7a). Enrichment results show that these predicted target genes participate in the jasmonic acid-mediated signaling pathway and response to salt and osmotic stresses (Supplementary Data 7b), in agreement with its previously validated function by Seo et al., 2011. We observed that OsbHLH148 is potentially involved in the regulation of ∼54% of genes in the module it is a part of (M0009; jasmonic-acid biosynthesis genes), indicating it acts as a hub in the local subnetwork. Additionally, we found 81 TFs among the predicted targets of OsbHLH148 and ∼82% of these TFs have more than one known bHLH binding site (5′-CANNTG-3′) within the 1000 bp upstream promoters (Supplementary Data 7c). This indicated that most predicted targets of OsbHLH148 are more likely to be downstream targets. Other TFs with no bHLH DNA-binding sites could be potentially be components of a larger co-activator complex. Among the predicted targets, we found three of the five TFs previously shown to interact with OsbHLH148 using Y2H assays (Seo et al., 2011). Among other predicted targets, OsRAP2.6 (also known as ERF101) and DREB1B TFs were most interesting because both these TFs are well-known to be critical regulators of stress responses in rice. DREB1B is a well-known TF previously shown to function in abiotic stress-responsive gene expression (Dubouzet et al., 2003). The OsRAP2.6 TF has been recently shown to regulate drought responses during rice’s reproductive development (Jin et al., 2018).

**FIGURE 9:**
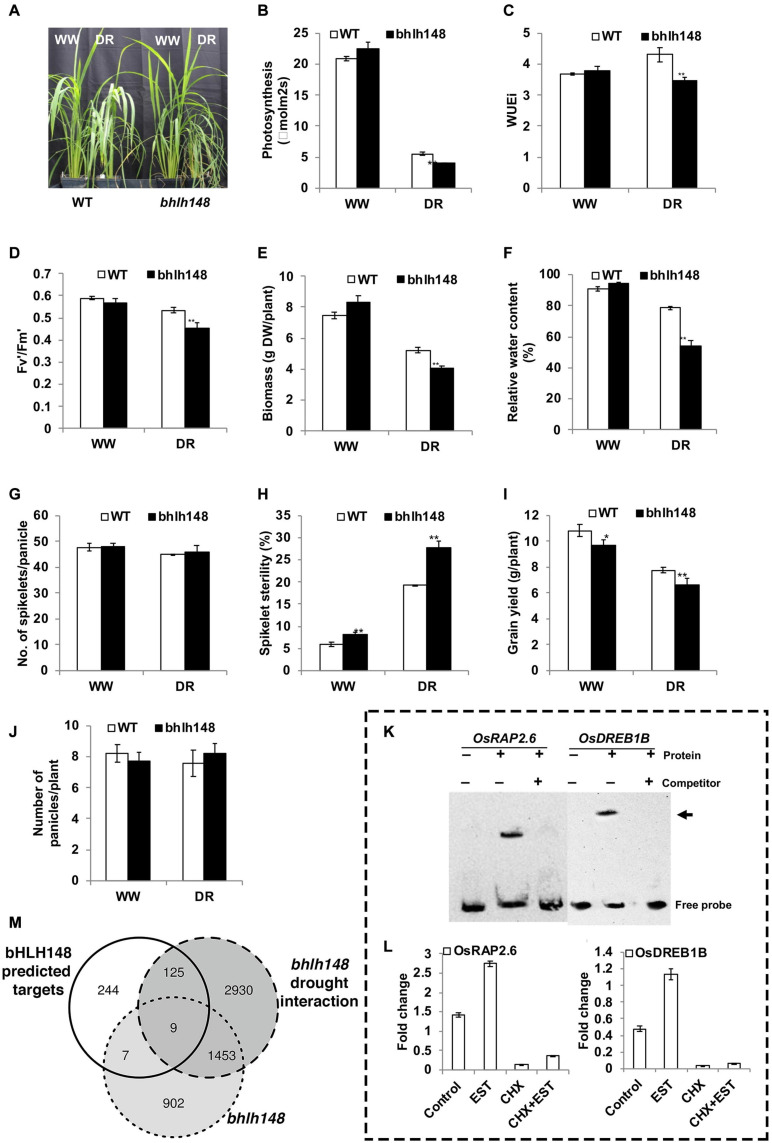
Phenotyping *bHLH148* as experimental validation of the GRAiN web application. OsbHLH148 was strongly predicted for association with drought by our network-based SVM classifier. We queried OsbHLH148 in the GRAiN web application and sought to test the predictions experimentally. **(A)** Increased sensitivity of *bhlh148* mutant plants under controlled drought stress conditions. Forty-five-day old plants were maintained at 100% (well-watered – WW) and 40% (drought – DR) FC (field capacity) for 10 days by a gravimetric approach, and performance was measured at the end of the stress period. **(B–F)** The phenotype of the WT and *bhlh148* mutant plants under drought stress. **(B)** Assimilation rate, **(C)** instantaneous water use efficiency (WUEi), **(D)** the efficiency of Photosystem II in light-adapted leaves, **(E)** above-ground biomass (dry weight), and **(F)** relative water content (RWC). Gas exchange measurements were taken using a portable photosynthesis system LI-6400XT at a CO_2_ concentration of 370 μmol/mol and light intensity of 1000 μmol/m^2^/s. The data are the means ± SE (*n* = 10) and significance using the *t-*test (**P ≤ 0.01). **(G–J)** Reduced grain yield of *bhlh148* plants under well-watered as well as drought stress conditions. Drought stress was applied by withholding irrigation at the R3 stage for 4–8 days until the leaves roll and wilt, followed by re-watering and maintaining under well-watered conditions until physiological maturity. Yield components were measured under well-watered and drought stress conditions at physiological maturity. **(G)** the number of spikelets, **(H)** percent spikelet sterility, **(I)** grain yield, and **(J)** the total number of panicles. The data are means ± SE (*n* = 6) and significance using *t-*test (*P ≤ 0.05 and **P ≤ 0.01). **(K,L)** Experimental validation of predicted OsbHLH148 targets predicted from the GRAiN web application. **(K)** Electrophoretic mobility shift assay (EMSA) was performed with bHLH148 protein and biotin-labeled promoter elements of potential bHLH148 regulated genes. bHLH148-6xHis recombinant protein was incubated with promoter elements at room temperature for 20 min. For competition analysis, the binding reaction was incubated for 10 min on ice before adding 100-fold excess of unlabeled promoter elements, followed by incubation at room temperature for 20 min. The samples were subjected to EMSA by PAGE and subsequent chemiluminescence detection. + and - indicate the presence and absence of the respective component in the binding reaction. Arrows indicate the labeled “free probe” and DNA-protein complex “bound probe” positions. **(L)** Direct activation of *OsRAP2.6* and *OsDREB1B* by bHLH148. Rice protoplasts were transfected with a bHLH148-HER fusion construct driven by the CaMV35S promoter. Transfected protoplasts were treated with estradiol (EST), cycloheximide (CHX), or EST and CHX together. The expression levels of *OsRAP2.6* and *OsDREB1B* in control and treated protoplast was analyzed by qPCR and shown for *RAP2.6* and *OsDREB1B*. Each data point is mean values ± SE of three biological replicates. **(M)** Venn diagram showing overlaps between GRAiN predicted targets of OsbHLH148 and genes that are differentially expressed in the mutant as well the mutant treated with drought.

Therefore, the prediction of OsRAP2.6 within the OsbHLH148 regulation raised a hypothesis that OsbHLH148 could also act as a regulator of drought responses during rice’s reproductive development. We acquired the homozygous loss-of-function knockout mutant line designated as ‘bhlh148’ to pursue this hypothesis. We performed extensive testing of this mutant’s phenotypes under controlled drought stress at the vegetative and reproductive stages. Under a well-watered (WW) condition, we found no significant phenotypic difference between the mutant and WT plants. However, under controlled drought stress treatment at 40% field capacity (FC), the mutant plants showed higher sensitivity with leaves rolled and collapsed than the WT plants (Figure 9A). Under drought, the *bhlh148* mutant plants showed a significant reduction in net photosynthetic rate, instantaneous water use efficiency (WUEi), the efficiency of Photosystem II measured in light-adapted leaves (Fv’/Fm’), above-ground biomass, and the relative water content (RWC) compared to WT (Figures 9B–F).

We applied drought stress to WT plants at the reproductive (R3) stage and observed a very high induction of OsbHLH148 in the inflorescence (3.1-fold) compared to flag leaf (0.55-fold) under drought stress relative to WW plants. The yield parameters for drought stress response, quantified by the number of spikelets/panicles (Figure 9G), spikelet sterility (Figure 9H), grain yield (Figure 9I), and the number of panicle/plant (Figure 9J), testify that OsbHLH148 is involved in grain yield under drought stress.

Next, we tested whether GRAiN correctly predicted the interaction of OsbHLH148 with OsRAP2.6 and DREB1B TFs. An electrophoretic mobility shift assay (EMSA) confirmed that bHLH148 binds to the promoters of OsRAP2.6 (LOC_Os08g36920) and OsDREB1B (LOC_Os09g35010) genes. We then used the steroid receptor-based inducible system to confirm that OsbHLH148 directly activates the expression of OsRAP2.6, while activation of OsDREB1B by OsbHLH148 requires additional factors (Figures 9K,L).

We also wanted to check if the other remaining genes predicted by GRAiN as targets of OsbHLH148 are correct. To confirm this, we performed gene expression profiling of bhlh148 and WT plants under WW and controlled drought stress conditions using mRNA sequencing (see Supplementary Methods). We used leaf tissue from plants maintained at 100 and 40% FC for 10 days as WW and controlled drought stress samples, respectively. We estimated the differential expression of genes that (1) responded to the knockout, and (2) responded specifically to the interaction of mutant with drought (subtracting the WT effect of drought from the mutant) (Supplementary Data 8). We found a relatively low overlap (<2%) between GRAiN predicted targets of OsbHLH148 and those significantly differentially expressed in the knockout. However, more than 32% of GRAiN predicted genes differentially expressed specifically due to bhlh148’s interaction with drought (Figure 9M). These observations testify that GRAiN naturally captures regulatory relationships that manifest specifically under stress rather than normal growth conditions.

### Experimental Support of the Predicted Drought Scores in the Literature

We re-scanned the literature to collect new TFs reported to be involved in DR phenotypes but published after we concluded our study. We found three such new DR TFs not included in our training data; OsHSFA3 (Zhu et al., 2020), OsMYB6 (Tang et al., 2019), and ONAC66 (Yuan et al., 2019). Our DR prediction model placed OsHSFA3 at rank 96 (decile 1), OsMYB6 at rank 376 (decile 2), and ONAC66 at rank 615 (decile 3), indicating that our model performs with great accuracy in the real-world.

## Conclusion

We integrated publicly available gene expression data of rice to infer an abiotic-stress response GRN. Because we used only microarray samples to create the gene expression matrix, our workflow was primed to be missing a considerable fraction of known rice genes not represented on the Affymetrix chip. This limitation could have been overcome by using RNA-seq datasets to assay a larger fraction of the genome. However, the number of RNA-seq datasets currently available to cover the broad spectrum of rice’s abiotic stress responses is limited. Using microarray chips allowed us to achieve a relatively larger sample-size while covering gene expression dynamics under various abiotic-stress treatments, growth stages, and cultivars.

Our study agrees with the previous reports which claimed that rather than using a single algorithm, an ensemble-centric approach improves the GRN inference performance. Adding diverse methods to an ensemble of network prediction methods should, theoretically, stabilize biologically relevant relationships between TFs and target genes (Marbach et al., 2012). We observed this phenomenon in our study, as the consensus predictions from the five network prediction algorithms outperformed individual methods (Figure 3). Interested researchers who wish to apply this consensus approach should also note that having more ensemble algorithms does not guarantee superior network inference performance. The correct combination of methods will depend on several factors, including the dimensions and the nature of the underlying dataset. In our study, removing the two correlation-based methods from the ensemble seems to have improved the final network’s performance in the experimental benchmark (ChIP-seq data) and not the two secondary reference networks (Figure 3). This could be explained by the fact that simple correlation-based methods are prone to a high accumulation of false positives arising from indirect correlations. The other three algorithms (CLR, GENIE3, and ARACNe) are specially designed to attenuate this problem. Using a consensus of only these three were better able to detect direct regulatory edges represented by ChIP-seq data. The *ad hoc* reference networks, on the other hand, might contain several false positives because they were built from derived data rather than direct experimental evidence. Therefore, removing the correlation-based methods from the ensemble barely made any difference when tested on this benchmark.

Several other algorithms not used in our study can potentially further improve the ensemble’s diversity. For example, module-based algorithms first apply clustering algorithms to the expression data and then assign regulators to the identified modules. While such an approach can potentially retrieve targets of TFs with less correlated expression profiles (De Smet and Marchal, 2010), there are several places in the module-based inference workflows where subjective biases can be introduced (e.g., the choice of the number of clusters to extract, which should ideally be chosen by thorough testing a range of clustering parameters). We found that module-based network inference algorithms generally have a more considerable computational burden (data not shown), especially on the relatively larger rice gene expression matrices. Other algorithms that use an integrative or supervised approach could also not be used in our study (Bonneau et al., 2006; Banf and Rhee, 2017; Zarayeneh et al., 2017). This is because the only other mutually exclusive datatype available for integration is the sequence-based DNA motif data. However, unlike expression patterns, most DNA binding motifs of rice TFs are not experimentally determined but predicted based on homologies. Also, a large fraction of rice TFs do not even have their corresponding binding sites predicted. Therefore, using DNA-motif data only to gauge the quality of the networks we predicted, but not the network inference itself, kept us in line with our goal of including as many genes as possible.

We named our network GRAiN. GRAiN is essentially a bipartite network as it has two types of nodes (TFs and modules). Our final goal was to develop an algorithm that uses machine learning to identify GRAiN patterns that characterize a particular set of nodes with verifiable attributes (gold standard TFs). To select our gold standard, we surveyed various phenotype databases. Our survey shows that while currently ∼2% (1098 at the time of this study) of all known rice genes have been linked to various abiotic stresses experimentally, more than 15% of these stress genes are TFs linked with drought or water deficit related responses. Our survey suggests that the genetic selection of favorable alleles of the stress-inducible TFs has been widely and inadvertently used as a tool to improve/select for drought tolerance. We listed 165 TFs linked with drought to train the machine learning algorithm. Our observations that most of these gold-standard drought regulators do not show sizeable differential expression patterns under drought further motivated us to develop such a computational model (Figure 4).

Our framework funnels an inferred modular GRN into the SVM that learned to discriminate between real drought TFs from those that are likely not regulators of drought, based on their network connectivity patterns. Our model’s application ranked every TF in the network according to their predicted association with DR. Therefore, the selection of regulatory genes using our approach remains less prone to subjective bias.

GRAiN and the subsequent network-based machine learning approach we presented in this study can also be applied to transcriptome collections within other biological contexts for which enough training labels are also available. Furthermore, the vertical integration of different data types could allow the development of more mechanistically informed models. Integrating GRAiN with other diverse sources of information (representing different layers of gene regulation) into a single prediction model will allow candidate gene selection in a truly holistic manner. For example, datasets featuring paired measurements of transcriptome networks (tissue or cell-type specific) and post-transcriptional regulation (e.g., small RNA-sequencing). Integration of other data types such as epigenetic profiles and post-translational modifications (PTM) such as phosphorylation also seems feasible with the SVM approach. Some excellent resources, such as the Plant PTM Viewer (Willems et al., 2019) and the database of phospho-sites in plants (Cheng et al., 2014) currently allow such data mining for a few plant TFs. Perhaps, vertical integration of heterogeneous data types could also help achieve a better classification of functional alleles in *indica* and *japonica* sub-types of rice, which remains a limitation of our study. However, the prediction models’ generalizability will depend upon the quality of training examples, the standard of validation data, and feature engineering.

In a nutshell, our study developed a novel computational framework for network-based prioritization of regulatory genes. Application of this pipeline accomplished three main challenges in rice: (i) identification of genes that participate in similar biological processes and pathways on the occurrence of abiotic-stresses, (ii) identification of genes co-regulated by a group of TFs, and (iii) prioritization of regulatory genes and modules associated with DR. We expect our drought prediction model to have superior performance in the real-world scenario, evident by the fact that three recently reported drought TFs, which we did not include in training our models, were correctly predicted. The network-based machine learning approach presented here, in conjunction with resources like the KitaakeX Mutant Database (Li et al., 2017), can support targeted screens to narrow down the search for TFs involved in specific physiological, morphological, and biochemical phenotypes to delineate specific DR mechanisms further. We anticipate that our study will be valuable for exploring the transcriptional regulatory code of stress responses in rice.

## Materials and Methods

### Development of the Consensus Gene Regulatory Network

A set of 35 Affymetrix microarray datasets comprising 265 individual gene expression samples under the context of abiotic stress were obtained from the gene expression omnibus (Supplementary Data 9). Datasets with at least four samples and two groups were retained, normalized, and processed into an integrated expression matrix. A comprehensive list of 2304 known rice genes annotated as TFs in several public databases was obtained (Yilmaz et al., 2009; Jung et al., 2010; Priya and Jain, 2013; Jin et al., 2014). This list of TFs and the gene expression matrix was supplied to five reverse-engineering algorithms. ARACNE was downloaded from the web link in the original publication. GENIE3 (Huynh-Thu et al., 2010) and CLR (Faith et al., 2007) runs were performed using the R package minet (Meyer et al., 2008). PCC and SCC were calculated using the Sleipnir library of functional genomics (Huttenhower et al., 2008). Note that all these algorithms are non-integrative and un-supervised. Meaning they aim to infer relationships between TFs and target genes solely from gene expression data (non-integrative), and without leveraging any prior knowledge of known interactions in the prediction process (un-supervised). Then, assuming no combinatorial regulation and feedback loops, ∼80 million regulatory links could have been predicted (35,151 genes X 2304 TFs) from the gene expression matrix. However, GRN inference remains an underdetermined problem, and knowing the exact number of true edges in a network is impossible (De Smet and Marchal, 2010). Therefore, to reduce the runtime of our workflow’s subsequent steps, we selected only the top 500,000 edges from each algorithm’s output (sorted and ranked based on the confidence metric given by the individual algorithm). Then, the union of selected edges from all algorithms was used to create an edge matrix *E*, with edges *i* in rows of *E* and algorithms *j* in columns of *E*. Each cell in the *E*_*ij*_ was populated by the rank given to *i* by *j*. Missing edges were substituted with the lowest rank of that column plus one (Marbach et al., 2012). The average rank of each row (edge) was then computed, and these averages were re-ranked to generate the final rankings. Hence, edges with small final ranks indicated greater confidence in all five methods. Edges with a final rank value of more than 500,000 were removed and the rest retained in the final consensus network.

### Creation of the ChIP-Seq Benchmark and Other Reference Networks

The ChIP-targets of 9 TFs were extracted from published data files with the original studies. The Position Weight Matrices (PWM) of ∼588 rice TFs listed in the CIS-BP database were obtained (Weirauch et al., 2014). PWMs indicate DNA sequence preferences of TFs and can infer DNA motifs in the promoter regions of functional genes. The 1000 bp upstream promoters were scanned for at least one or more PWM motifs using the FIMO tool in the MEME suite (Bailey et al., 2015). Motifs found in more than 50% of all the genes were treated as ‘constitutive elements’ and removed. Genes harboring all the remaining motifs with a *p*-value < 1E-10 were linked to the corresponding TFs. The GO BP reference was created by using evidence of functional relationships between TFs and non-TF genes co-annotated in the rice biological process (BP) ontologies. Only those annotation labels consisting of less than 200 genes were chosen for this. Excluding large BPs ensured that minimally related genes (in processes such as ‘translation,’ ‘DNA repair,’ ‘signal transduction’ etc.) did not become part of the reference network. The PPI reference network of rice was obtained from the PRIN database (Gu et al., 2011) hosted at http://bis.zju.edu.cn/prin/download.do. Only experimentally verified interactions were used, and edges with at least one TF as a corresponding node were identified.

### Finding Modules in the Gene Regulatory Network

Note that the network structure obtained by taking the consensus of the ensemble was essentially a mixed bipartite graph. One set of nodes represented TFs, and the other set of nodes represent target genes (functional genes plus TFs). To detect modules in such a network, the graph’s biadjacency matrix was first converted to a unipartite network following the approach used to build the Arabidopsis stress network (Vermeirssen et al., 2014). Then, the similarity in predicted regulators of every pair of genes was estimated using the JI of overlap. Operationally, this technique accounts for the dogma of co-regulation instead of co-expression, thus preserving the network’s regulatory nature. Then, we applied the Markov clustering algorithm (mcl) on this network to find modules of co-regulated genes. The inflation parameter of the mcl algorithm was set to a value of 2 after tuning (data not shown).

### Functional Annotations of Network Modules

Annotations in the rice GO, the KEGG, and CYC pathways were obtained from the plant GSEA server (Yi et al., 2013). The mapman annotation file was obtained from the MapManStore^2^. GO annotations were propagated from parent terms to child terms using the true-path rule, as described before (Ambavaram et al., 2014). From all these databases, categories that annotate more than 500 genes and less than three genes were removed. Statistical significance of overlaps between remaining gene-sets and co-regulated gene modules was calculated using hypergeometric tests. The resulting *p*-values were corrected for multiple testing using the Benjamini–Hochberg method (Benjamini and Hochberg, 1995). A *q*-value threshold of 0.05 was set to declare an observed enrichment as significant.

### *De novo* Analysis of CREs

The FIRE tool was supplied with 1000 bp upstream promoter sequences of genes within each module identified by the mcl algorithm. The parameter *k*, which defines the length of seed sequences, was kept between 3 and 12, and only DNA motifs were inferred. Only those motifs that occur within at least 50% of each module’s genes were selected for all FIRE runs. These motifs were then matched against plant CREs listed in various public databases using the STAMP server (Mahony and Benos, 2007). Matching CREs with a *p-*value less than 0.001 were tagged as associated with the corresponding module. FIRE detected motifs were converted to meme format and matched with DAP-seq motifs from Arabidopsis using the tomtom tool in the meme suite (Bailey et al., 2015). CIS-BP motifs were similarly compared.

### Development of the GRAiN Application

The GRAiN web application is open source^3^. The code is written in the R programming language (R Core Development Team, 2012) and runs on the Shiny platform^4^. The application uses the piano package (Väremo et al., 2013) to estimate the statistical significance of overlaps between a query geneset (user entered list of genes or retrieved TF targets) and a collection of genesets (modules or GO BP sets). All genesets with the Fisher’s exact test FDR corrected *p-*value < 0.05 are returned to the user. Network data is processed using the igraph library (Csardi and Nepusz, 2006) and visualized using the visNetwork package^5^.

### Generating Examples for Machine Learning

The gene keyword file from the funRiceGenes server was obtained https://funricegenes.github.io/ in May 2019. Gene lists available in the Oryzabase database were obtained from https://shigen.nig.ac.jp/rice/oryzabase/download/gene on the same day. The rice mutant database was obtained from the published article (Zhang et al., 2006). Using a word cloud analysis (not shown), most prominent keywords in these databases were visualized. Genes linked with keywords related to abiotic stress such as “drought,” “water-deficit,” “salt,” “cold,” “heat,” and “temperature” were then extracted. The retrieved locus IDs and publication records of genes were manually scanned for consistency by expert stress biologists, and TFs linked with drought (and related keywords) were labeled as positives. Note that OsbHLH148 was initially present in our dataset as a drought positive TF (Seo et al., 2011). However, we removed it from the positive list before training the models as a hidden example on which wet-lab experiments were performed later. We listed negative examples from the remaining TFs as those that were not positive for any abiotic stress (salt, cold, and heat), since many genes are multi-stress responsive. We also reanalyzed seven published gene expression datasets covering drought stress responses in various organs and tissues of rice plants across multiple genotypes. TFs that did not differentially express in these datasets were also counted as drought negatives. In addition to this, the rice stress TF database was downloaded (Priya and Jain, 2013) from http://www.nipgr.ac.in/RiceSRTFDB.html and TFs not listed as responsive to drought and salt in this database was also included as negative TFs. Altogether, we created a pool of 752 TFs that are most likely not regulators of drought stress responses.

### Network-Based Classifier

GRAiN is structured as a matrix *G*, with each entry in *G*_*ij*_ corresponding to the JI of TF *i* in a row with module *j* in the column. *G* was supplied as the feature-set to the linear kernel SVM classifier. The vector of JI values of each labeled TF across all modules in *G* represented its feature vector. The objective of an SVM function is to identify the best hyperplane that separates the two classes of the training data (drought positive and negative TFs) using their feature vectors. The width of the margin that separates the two classes was controlled by optimizing the classification trade-off parameter (*C*; a penalty for a miss-classified example). An optimal *C* = 1 was chosen by testing a range of values from 0.001 to 10 in increments of 0.1 and five-fold cross-validation tests. This test split all training examples (drought positive and negative TFs) into five equal parts. The model was trained on four of the five splits and tested on the remaining split kept hidden in training, ensuring that each split was used as the test-set only once. Model accuracy was evaluated using ROC statistics. Classifier training and learning were performed using the SVMperf implementation in the Sleipnir library. Cross-validation splits and performance evaluation was performed using the ROCR (Sing et al., 2005) and PRROC packages in R. The SVM returned distance from the hyperplane *D* for each TF in GRAiN. The values of *D* were averaged over four values from the final five-fold cross-validation test and then scaled over a range from 0 to 1. The resulting value of each TF was referred to as its DS.

### Analysis of RNA-Seq Data and Estimation of Differential Expression

Raw fastq files of individual samples from all external datasets were downloaded from the SRA. The Nipponbare RefSeq (MSU version 7) was obtained from the rice genome annotation project website (Kawahara et al., 2013). The barley and sorghum genomes and annotations were downloaded from the Phytozome web portal (Goodstein et al., 2012). The following procedure was uniformly applied across all RNA-seq samples, including samples from mutant experiments generated in the study described here. Reads were mapped to the respective reference genomes using STAR version 2.7 (Dobin et al., 2013). The bam files obtained from STAR runs we sorted using samtools and used as input to the HTseq software version 0.11.2 (Anders et al., 2015) with its default parameters for counting reads per gene per sample. Count of reads obtained from HTseq runs were then integrated as a count matrix (one for each experiment) with columns representing individual samples and rows representing genes. Each cell of the matrix presented raw counts of the gene in the corresponding sample. Each gene’s count was first scaled by its length to give reads per kilobase (RPK). The sum of all RPK values per sample divided by 1 million gave us a scaling factor. Then, dividing each RPK value by this scaling factor computed gene expression as transcripts per million (TPM) units. The effective gene length to be used in RPK values calculations was calculated as the sum of non-overlapping exon lengths using the genomic features package in R (Lawrence et al., 2013). The GFF3 files of all genomes were converted to GTF format using GFF utilities (gffread) of the cufflinks software (Trapnell et al., 2010). The resulting GTF file was used as input to genomic features for effective gene length calculation. Note that the rice GFF3 file on rice MSU reference has mis-annotations of ∼1000 gene isoforms, which hampered gene length calculations. Conversion of GFF3 to GTF ensured proper grouping of individual transcripts to parent gene ID. For the test of differential expression, the raw count data was normalized using edgeR (Robinson et al., 2009) and transformed using voom (Law et al., 2014). The voom-transformed values were used for linear modeling using the limma package in R (Ritchie et al., 2015). Differential expression of genes between control and treatment samples was estimated from the coefficients of the linear models. The interaction between *bhlh148* and drought was estimated by subtracting the baseline effect of drought on the WT sample from the effect of drought on the mutant sample (on a log scale). Differential expression from each microarray dataset was calculated as follows. Each dataset was background corrected, normalized, and summarized using the RMA algorithm (Irizarry et al., 2003). Then, genes with interquartile range across samples less than the median interquartile range were filtered. A linear model was then used to detect the remaining genes’ differential expression using limma, as described above. In all cases, *p*-*values* were converted to *q-values* using the qvalue package in R to account for multiple hypothesis testing.

### Controlled Drought Stress at Vegetative Stage and Physiological Measurements in Rice

To test the drought stress response of mutant plants at the vegetative stage, we applied controlled drought stress on 45-day-old plants using a gravimetric approach. One-week old equal-sized individual seedlings were transplanted into 4 square inch plastic pots filled with Redi-earth potting mix of a known weight and water holding capacity. Thirty-five days after transplanting, controlled drought stress (DR) was initiated on ten pots and monitored gravimetrically. The soil water content was brought down to 40% FC for 3–4 days, and plants were maintained at that level for 10 days by weighing the pots daily at a fixed time of the day and replenishing the water lost through evapotranspiration. Another ten pots were maintained at 100% FC and treated as WW conditions (Ramegowda et al., 2014). At the end of the stress period, gas exchange and light-adapted fluorescence measurements (Fv’/Fm’) were taken on the 2nd fully expanded leaves from the top, using a portable photosynthesis meter, LI-6400XT (LI-COR Inc., NE, United States) at a CO_2_ concentration of 370 μmolmol^–1^, the light intensity of 1000 μmolm^–^2s^–1^ and RH of 55–60%. Instantaneous WUEi was calculated using the net photosynthetic rate (A) and transpiration rate (T) as WUEi = (A/T). Leaf RWC was measured as described (Barr and Weatherley, 1962) in the leaves used for gas exchange measurements. The leaf fragments of the same length were excised, and fresh weight (FW) was measured immediately. Leaf fragments were hydrated to full turgidity by floating them on deionized water for six h, then blotted on a paper towel and the fully turgid weight (TW) taken. The leaf samples were then oven-dried at 80°C for 72 h and weighed to determine the dry weight (DW). The percent RWC was calculated as RWC (%) = (FW – DW)/(TW – DW) × 100. Shoots were harvested, oven-dried at 80°C for 72 h, and weighed to determine biomass.

### Grain Yield Analysis Under Reproductive Drought in Rice

The effect of drought stress on grain yield of the rice genotypes was tested by applying drought stress to plants at the R3 stage (Counce et al., 2000). Individual plants in 4 square inch plastic pots were grown at WW conditions until the R3 stage. Drought stress was applied by withholding water at the R3 stage for 4–8 days until all of the leaves wilted, followed by re-watering. Panicles exposed to drought stress during the 4–8 days window were marked and used for yield component analysis. A set of WW plants were also maintained as controls. Plants were further grown in WW conditions until physiological maturity. Drought exposed panicles were harvested, and the number of filled and unfilled spikelets counted to determine spikelet sterility (%). The filled spikelets were dried at 37°C for 5 days and weighed to determine grain yield/plant.

### Electrophoretic Mobility Shift Assay (EMSA)

The total RNA isolated from drought-stressed rice plants was used to amplify full-length cDNA encoding bHLH148 and cloned into pET28(a) vector at *Bam*HI and *Eco*RI sites. The bHLH148-6xHis recombinant fusion protein expression was induced with 1 mM IPTG for 4 h and purified using Ni-NTA resin. The identity of the purified protein was confirmed by western blotting (data not shown) using the His-tag antibody. The binding reaction and EMSA were carried out using a standard protocol according to the manufacturer’s instructions (LightShift Chemiluminescent EMSA Kit). Promoter sequences (2 kb upstream of transcription start site) of AP2/ERF TFs were identified using the PlantPAN database^6^ (Chang et al., 2008) and searched for the presence of E-box elements in the PLACE database^7^ (Higo et al., 1999). Specific sets of primers were used to amplify 200 bp E-box flanking regions of each of the putative bHLH148-regulated gene promoters using rice genomic DNA as a template. The amplified promoter fragments were biotin-labeled at the 3′ end using the Biotin 3′ End DNA Labelling Kit (Pierce). The binding reactions were carried out in a buffer containing 10 mM Tris (pH 7.5), 50 mM KCl, 1 mM dithiothreitol, 2.5% glycerol, 5 mM MgCl, 0.05% Nonidet P-40, and 50 ng/μl of poly(dI-dC). For competition analysis, the binding reactions were incubated for 10 min on ice before adding 100-fold excess of unlabeled competitor DNA, and the reaction mixture was further incubated for 20 min at room temperature before loading onto a 5% native polyacrylamide gel. The resolved DNA-protein complexes were electro-blotted onto nylon membranes and subsequently detected using the chemiluminescence detection kit.

### Steroid-Inducible System for Testing Direct Activation of Genes by bHLH148

The bHLH148-HER expression construct was generated by ligating the PCR-amplified full-length cDNA of bHLH148 at the *Kpn*I site fused with the regulatory region of HER at the C terminus between the CaMV 35S promoter and the NOS terminator in pUC19 vector. The construct was transfected into rice protoplasts by electroporation and incubated with 2 μM estradiol for 6 h to release cytoplasmic bound bHLH148. For the control reactions, the same concentration of ethanol used to dissolve estradiol was used. Protoplasts were treated with cycloheximide (2 μM) for 30 min before the addition of estradiol to inhibit new protein synthesis. Total RNA was isolated from the treated protoplasts and used for qPCR analysis. The data presented are the averages of three biological replicates.

All primers used in this study can be found in Supplementary Table 2.

## Data Availability Statement

New RNA-seq datasets generated in this study can be found in NCBI GEO online repository (https://www.ncbi.nlm.nih.gov/geo/) under the accession GSE65024.

## Author Contributions

CG and AP conceived the idea. CG executed it, developed the web application, and drafted the manuscript. CG, VR, and AP designed the experiments. VR performed the drought assays and physiological analysis. SB performed the interaction experiments. AP acquired the funding and supervised the research. All the authors contributed to writing the manuscript.

## Conflict of Interest

The authors declare that the research was conducted in the absence of any commercial or financial relationships that could be construed as a potential conflict of interest.

## Footnotes

1 http://rrn.uark.edu/shiny/apps/GRAiN/

2 https://mapman.gabipd.org/mapmanstore

3 https://github.com/cngupta/GRAiN

4 https://shiny.rstudio.com/

5 https://datastorm-open.github.io/visNetwork/

6 http://plantpan.itps.ncku.edu.tw/

7 http://www.dna.affrc.go.jp/PLACE/

## Supplementary Material

Supplementary Figure 1 Analysis of *cis*-regulatory elements within coregulated modules. **(A)** A bar plot showing the proportion of co-regulated modules (*x*-axis) containing **a** differing number of *cis*-regulatory elements (CREs) (*y-*axis). **(B)** A waffle plot (alternative to a circular pie chart) showing the fraction of *de novo* identified CREs that matched different sources (colored uniquely) of putative plant CREs.

Supplementary Figure 2 A network analysis of novel *cis*-regulatory elements. Besides recovering several known plant CREs, FIRE identified three novel motifs that did not match to any known plant CRE. The heatmap shows that these novel motifs could potentially be direct or ‘associative’ binding sites of members from seven TF families, based on significant overlaps of the predicted targets of TFs from the families on the *x*-axis within the genes that harbor the three novel CREs on the *y* axis (FDR-corrected hypergeometric tests *p*-value < 0.01). The color gradient indicates the network score, calculated as the average ranks of edges from the consensus gene regulatory network. Darker color indicates a stronger association between the CRE and the TF family, as indicated in the key.

Supplementary Figure 3 A line plot showing relationships between the predicted drought score and network degrees of TFs.

Supplementary Figure 4 A subset of modules with the highest feature importance scores from the drought classifier were connected to *cis*-regulatory elements (CREs; predicted by *de novo* analysis) found enriched within them, as well as to their predicted regulators (TFs). The regulators were, in turn connected to the CREs based on enrichment analysis (FDR corrected hypergeometric test *p*-value < 0.01). This interconnected network with three node types (modules, CREs, TFs) was visualized in Cytoscape (version 3.0). Modules are indicated in rounded rectangles, CREs in ellipses and TFs in triangles colored according to the family membership indicated in the key on the right. The network shows hubs of different node-types.

Supplementary Figure 5 Predicted drought scores in relation to the pan-genome of rice. Boxplots showing drought score distributions of **(A)** core TF, **(B)** indica dependent TFs, and **(C)** japonica dependent TFs.

Supplementary Table 1 Evaluation of different network inference methods. Algorithms were tested for their ability in recovering putative cis elements listed in CIS-BP database in the promoters of their predicted target genes.

Supplementary Table 2 Primers used in the study.

Supplementary Data 1 Top 500,000 edges inferred by the ensemble and their aggregate.

Supplementary Data 2 Gene-module memberships.

Supplementary Data 3 Module CRE annotations.

Supplementary Data 4 Module pathway/process annotations.

Supplementary Data 5 Drought scores.

Supplementary Data 6 Feature importance scores.

Supplementary Data 7 OsbHLH148 GRAiN prediction results.

Supplementary Data 8 OsbHLH148 differential expression test results.

Supplementary Data 9 GEO datasets used to build GRAiN.
