# Supplementary figures and images for: Using Network-Based Machine Learning to Predict Transcription Factors Involved in Drought Resistance
Source: Front Genet. 2021 Jun 24;12:652189. doi: 10.3389/fgene.2021.652189 (PMC8264776; doi:10.3389/fgene.2021.652189)

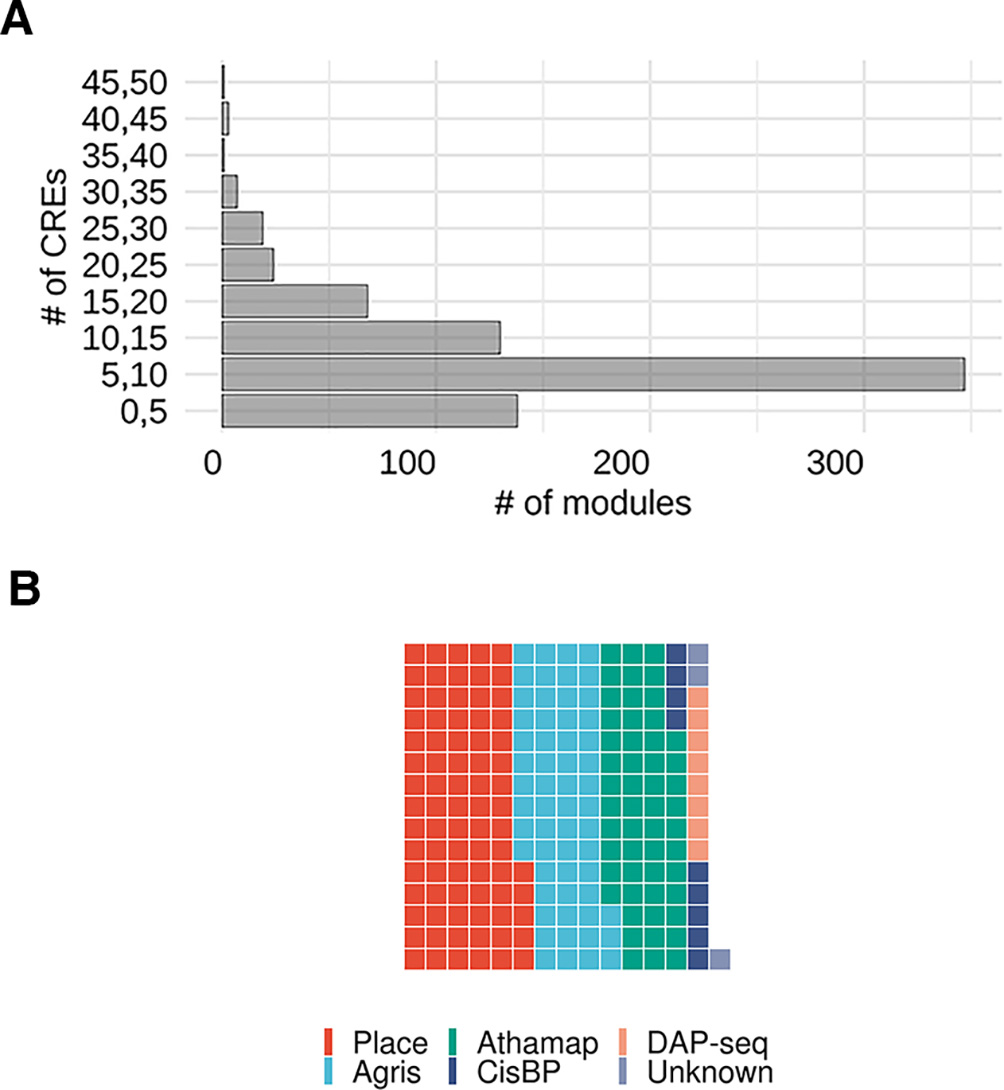

Supplement: Supplementary Figure 1 — Analysis of cis-regulatory elements within coregulated modules. (A) A bar plot showing the proportion of co-regulated modules (x-axis) containing a differing number of cis-regulatory elements (CREs) (y-axis). (B) A waffle plot (alternative to a circular pie chart) showing the fraction of de novo identified CREs that matched different sources (colored uniquely) of putative plant CREs. [file Image_1.JPEG]

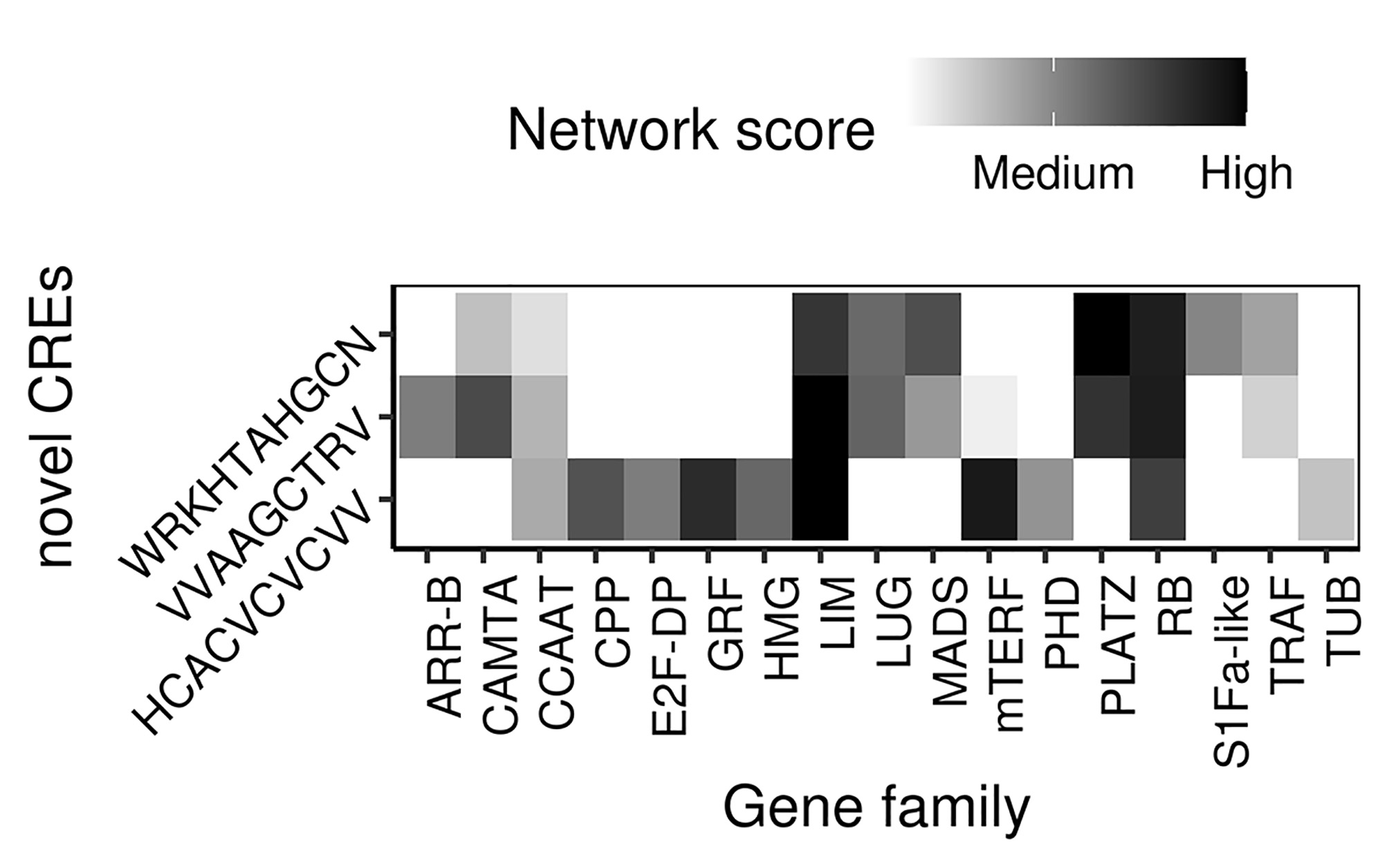

Supplement: Supplementary Figure 2 — A network analysis of novel cis-regulatory elements. Besides recovering several known plant CREs, FIRE identified three novel motifs that did not match to any known plant CRE. The heatmap shows that these novel motifs could potentially be direct or ‘associative’ binding sites of members from seven TF families, based on significant overlaps of the predicted targets of TFs from the families on the x-axis within the genes that harbor the three novel CREs on the y axis (FDR-corrected hypergeometric tests p-value < 0.01). The color gradient indicates the network score, calculated as the average ranks of edges from the consensus gene regulatory network. Darker color indicates a stronger association between the CRE and the TF family, as indicated in the key. [file Image_2.JPEG]

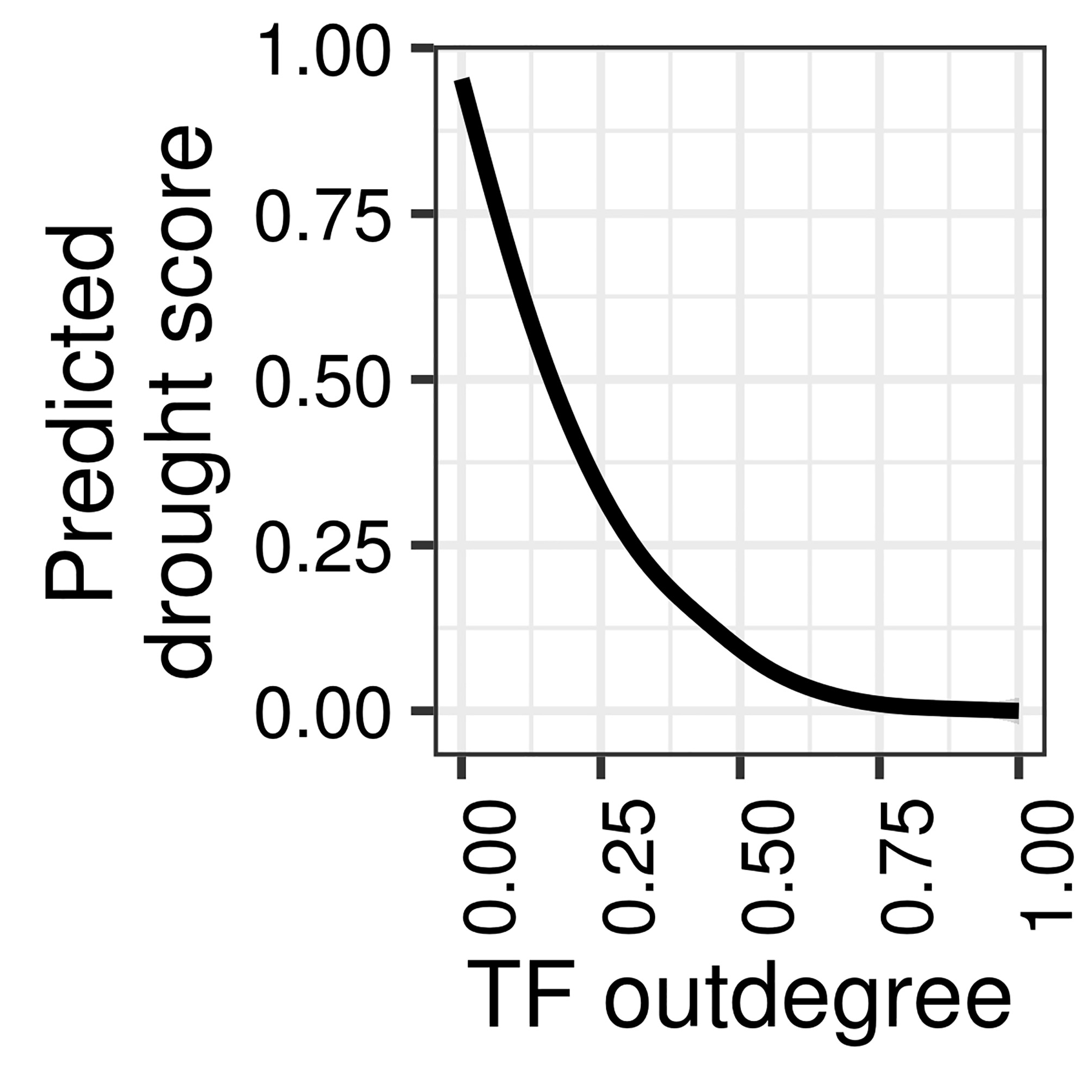

Supplement: Supplementary Figure 3 — A line plot showing relationships between the predicted drought score and network degrees of TFs. [file Image_3.JPEG]

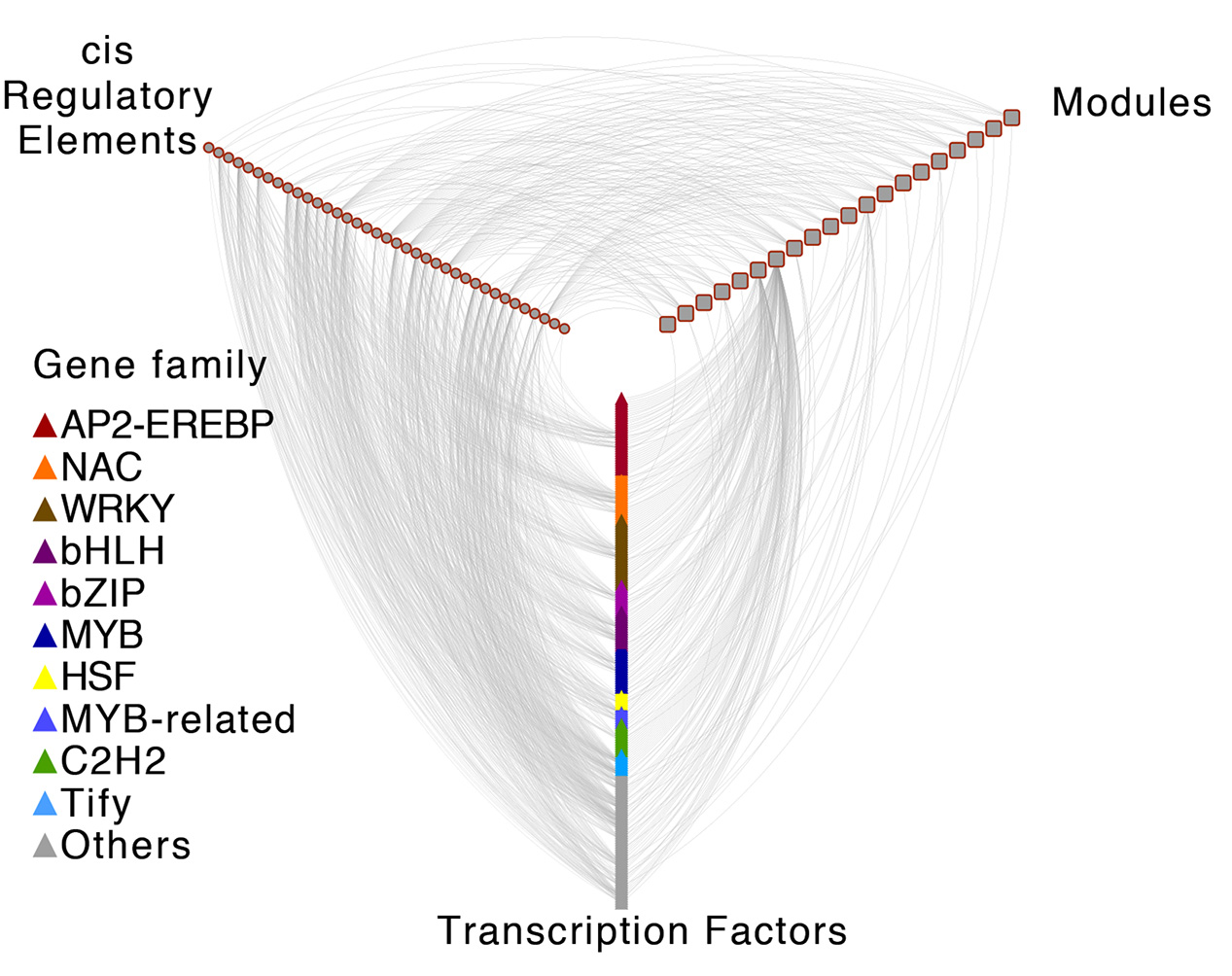

Supplement: Supplementary Figure 4 — A subset of modules with the highest feature importance scores from the drought classifier were connected to cis-regulatory elements (CREs; predicted by de novo analysis) found enriched within them, as well as to their predicted regulators (TFs). The regulators were, in turn connected to the CREs based on enrichment analysis (FDR corrected hypergeometric test p-value < 0.01). This interconnected network with three node types (modules, CREs, TFs) was visualized in Cytoscape (version 3.0). Modules are indicated in rounded rectangles, CREs in ellipses and TFs in triangles colored according to the family membership indicated in the key on the right. The network shows hubs of different node-types. [file Image_4.JPEG]

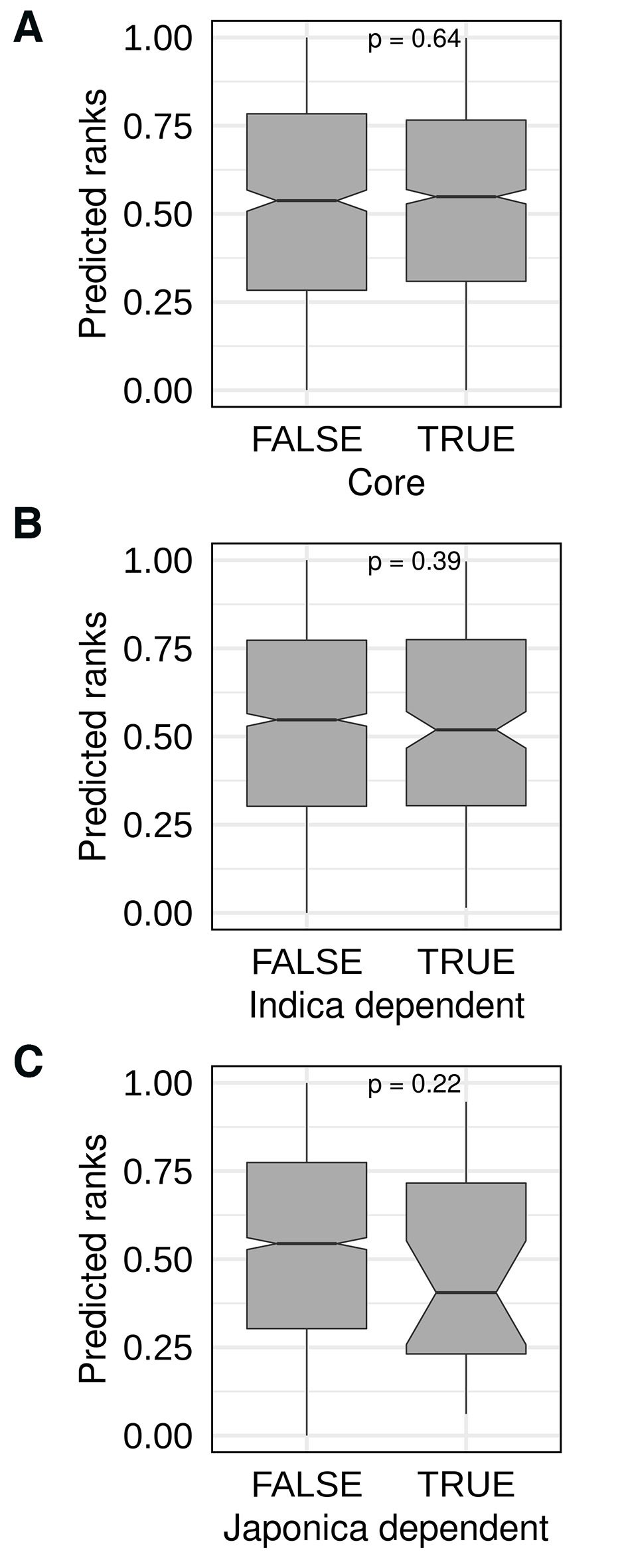

Supplement: Supplementary Figure 5 — Predicted drought scores in relation to the pan-genome of rice. Boxplots showing drought score distributions of (A) core TF, (B) indica dependent TFs, and (C) japonica dependent TFs. [file Image_5.JPEG]
