# Supplementary material for: Using Network-Based Machine Learning to Predict Transcription Factors Involved in Drought Resistance
Source: Front Genet. 2021 Jun 24;12:652189. doi: 10.3389/fgene.2021.652189 (PMC8264776; doi:10.3389/fgene.2021.652189)
Supplement: Supplementary Table 2 — Primers used in the study. [file Table_2.docx]

Supplemental Table S2: Primers used in the study

| **Primes used for genotyping *bHLH148* mutant in rice** | | |
| --- | --- | --- |
| bHLH148F | CGTAGTGGCAACGGGATAAC |  |
| bHLH148R | ATAGCAGCAGGCCTGAAGAA |  |
| T-DNA primer | GTCTGGACCGATGGCTGTGTAGAAG |  |
| **Primes used for expression analysis of *bHLH148*** | | |
| bHLH148q F | ATGCAAATGGAGTCGTACTAC |  |
| bHLH148q R | TCAAAACACATTTTGCACATGAGATG |  |
| **Primes used for expression analysis of AP2 transcription factor genes** | | |
| OsRAP2.6 F | AAGCTCAACTTCCCGTTCC |  |
| OsRAP2.6 R | AGACAATGTGTCGGACTTGG |  |
| OsDREB1B F | GATGGCGACGAAGAAGAAGA |  |
| OsDREB1B R | GAACCTGAACCCGTCGTC |  |
| **Primers used to amplify promoter elements for EMSA** | | |
| OsRAP2.6p F | AAATAAATAAGACAGACGATT |  |
| OsRAP2.6p R | TGGAACTTTTTCAGCCAT |  |
| OsDREB1Bp F | GGAGGAGTAAACCGCGA |  |
| OsDREB1Bp R | AACGGTGTGTTTTTTTAT |  |
