# Supplementary material for: Using Network-Based Machine Learning to Predict Transcription Factors Involved in Drought Resistance
Source: Front Genet. 2021 Jun 24;12:652189. doi: 10.3389/fgene.2021.652189 (PMC8264776; doi:10.3389/fgene.2021.652189)
Supplement: Supplementary file 17 [file Presentation_1.PDF]

Supplemental Methods by Chirag Gupta, Venkategowda Ramegowda, Supratim Basu and Andy Pereira for “Using network-based machine learning to predict transcription factors involved in drought resistance”

Rice growth conditions and stress treatments

Oryza sativa ssp. *japonica* cv. Nipponbare was used in the study. Unless otherwise mentioned, rice plants were grown in the greenhouse conditions with 26°C ± 1 day /22°C ± 1 night temperature, 600 $\mu molm^{-2}s^{-1}$ light intensity and 14 h light and 10 h dark cycles. Plants were grown in pots filled with Redi-earth potting mix (Sun Gro Horticulture Distribution Inc., Bellevue, WA, USA), placed in water-filled trays to simulate flooded condition and fertilized every week with 24-8-16 Miracle-Gro (Scotts Miracle-Gro Products, Inc., Marysville, OH, USA) until used for the experiment. The *bhlh148* T-DNA insertion line (Plant ID: ATOG05) was identified at the Oryza Tag Line (OTL) Database (http://orygenesdb.cirad.fr/tools.html) and seeds from mutant plants were obtained from CIRAD (Droc et al., 2006). The homozygous mutant plants were identified by PCR analysis using a pair of gene-specific primers flanking the insertion site and a combination of gene-specific and T-DNA border primers.

Transcriptome sequencing

Total RNA from leaf samples of WT and *bhlh148* plants grown under controlled drought stress (40% FC) and well-watered (100% FC) conditions was isolated using TRIZol reagent (Invitrogen) following the manufacturer's instructions. Total RNA was treated with RNase-free DNase I (Promega) and passed through RNeasy spin columns (Qiagen) to remove DNA and other contaminants. The concentration, quality and integrity of the RNA were analysed using the Agilent 2100 bioanalyzer (Bio-Rad) and NanoDrop™ ND-1000 (Thermo Scientific). Samples with RNA Quality Indicator (RQI) of > 8.0 were used for RNA-sequencing. Two biological replicates per sample were analysed. The cDNA library was constructed using TruSeq Stranded total RNA with Ribo-Zero Plant kit (Illumina). Sequencing was carried out on each library to generate

50 bp SE reads using Illumina High-Seq 2000 platform (Iowa State University DNA Facility).
